# Principles and Practical Measurement and Control Strategies for Achieving Ultrahigh CO_2_ Capture Rates in Amine Post-Combustion Capture Plants

**DOI:** 10.1021/acs.iecr.6c02191

**Published:** 2026-09-23

**Authors:** Lucas Joel, Aisha Ibrahim, Daniel Mullen, Abdulaziz Gheit, Muhammad Akram, Changyou Xia, Xi Liang, Qiang Jing, Weiming Zhao, Mathieu Lucquiaud, Jon Gibbins

**Affiliations:** † School of Mechanical, Aerospace and Civil Engineering, 7315 The University of Sheffield, Sheffield, South Yorkshire S3 7RD, United Kingdom; ‡ SSE Thermal, One Forbury Place, 43 Forbury Road, Reading RG1 3JH, United Kingdom; § Translational Energy Research Centre, The University of Sheffield, Sheffield S9 1ZA, United Kingdom; ∥ UK-China (Guangdong) CCUS Centre, 1 Tianfeng Road,Science City, Guangzhou 510520, China; ⊥ Shenzhen Shenshan Special Cooperation Zone China Resources Power Co., Ltd, Shenzhen 518066, China

## Abstract

This study presents a practical feedback control strategy for conventional amine post-combustion capture (PCC) plants that is capable of achieving up to around 100% added CO_2_ capture (i.e., the exit CO_2_ is equivalent to the CO_2_ in the combustion air) with moderate energy inputs. It has been demonstrated using MEA, but the fundamental principles appear to be more widely applicable. The method involves controlling the desorber to produce the lowest possible lean loading without excessive energy input using simple temperature measurements and then closely regulating the liquid-to-gas ratio in the absorber so that the minimum amount of solvent required to achieve the target capture rate is used. The principles behind the control method are illustrated using process modeling, and its practical viability is shown by a successful 75 h continuous period of controlled ∼100% capture in pilot testing at 30 tCO_2_/day scale. Implementation was found to require only minimal operator training and was able to be accomplished using standard plant instrumentation supplemented by a flue gas analyzer suitable for the resulting ∼500 ppmv exit CO_2_ levels. The control approach therefore appears transferable to commercial amine capture facilities, although maximum achievable capture rates will depend on a plant’s physical design. This paper provides actionable insights for plant design, particularly key instrumentation provisions, and supports permitting guidelines for regulators.

## Graphical abstract

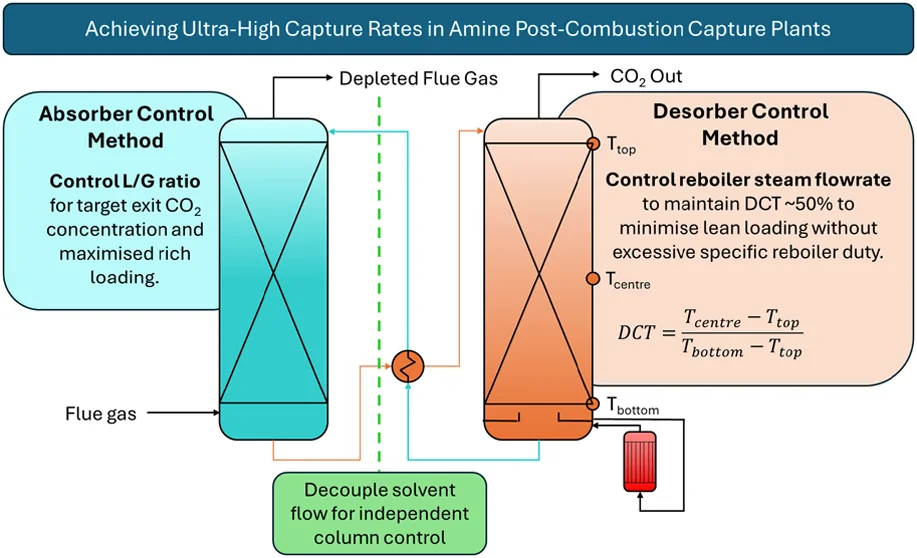

## 1 Introduction

Amine post-combustion capture (PCC) plants are currently being chosen for the majority of commercial flue gas CO_2_ capture projects.^1^ Because global CO_2_ emissions need to reach net zero, and eventually net negative, in order to avoid dangerous climate change,^2^ it is important that, even in these first phases of rollout, PCC plants are designed and operated to achieve very high capture rates, both because they are likely still to be operating when net zero targets are in place and also to give relevant learning to underpin future generations of the technology.

Net zero operation requires, among other things, that any residual fossil CO_2_ leaving a capture unit has to be recaptured from the atmospheric air. The cost for doing this carbon dioxide removal (CDR), compared to the cost of avoiding the residual emissions at source, is what sets the theoretically optimum capture rate, but future CDR costs must be estimated and actual costs for achieving a particular capture rate depend on plant-specific details and will also vary dynamically with time as other costs and revenues associated with plant operation (e.g., fuel cost, electricity price) vary. Calculating the optimum design capture rate for a specific project therefore requires at least the level of information available in a FEED study and, even then, operators will continually reassess the optimum capture rate that the plant should achieve over the plant’s lifetime depending on the various market factors that then apply, possibly as frequently as every hour, e.g., as electricity market prices change.

It is not the purpose of this paper to attempt to predict theoretical required capture rates under various assumed future scenarios. Rather, we report the principles and initial practical trials behind a feedback control method able to maintain, as conditions vary, continuous operation at high capture rates, up to around 100% added fuel CO_2_ capture, with minimum solvent regeneration energy requirements. A 100% capture rate on this basis means that the CO_2_ in the flue gases leaving the plant is equal to the amount that came in with the combustion air, giving zero direct CO_2_ emissions and thus removing the need for additional CDR. Table 1 shows, for a range of applications, examples of the exit flue gas CO_2_ concentrations at 100% added CO_2_ capture and also the corresponding rate of capture based on the total inlet flue gas CO_2_ concentration.

**Table 1:** Examples of Approximate Absorber Exit Dry CO_2_ Concentrations for 100% Added CO_2_ Capture and Corresponding Total Inlet CO_2_ Capture Rates

flue gas	assumed empirical formula for the fuel	excess air [%]	flue gas CO_2_ concentration including atmospheric excess air (%v/v dry)	calculated absorber exit CO_2_ with 100% added CO_2_ capture [ppmv]	% of total CO_2_ flue gas captured
coal	CH_0.81_O_0.08_S_0.02_N_0.01_ ^3^	15 ^4^	15.92	513	99.68
CCGT	CH_4_	168.4	4.2 ^5^	466	98.89
BECCS	CH_1.4_O_0.6_ ^6^	30 ^7^	15.55	501	99.68
WECCS	C_32_NH_55_O_16_ ^8^	190 ^9^	6.25	452	99.28

Traditionally, arbitrary capture rates of 90% of the total flue gas CO_2_, or even 85% for combined cycle gas turbine (CCGT) flue gases with lower CO_2_ levels, have been specified in standardized studies commissioned by bodies such as the IEAGHG (e.g., see study parameters in ref 10), where the studies’ main purpose has been to compare different capture technologies. This has led, intentionally or otherwise, to the use of plant design and operating parameters that are adequate, and to some extent optimized, for these capture rates but that prevent effective operation at higher capture rates. Many energy system modeling studies have also assumed that, e.g., a 90% capture rate is the limit for CCS plants in general, e.g., ref 11, with significant limits then imposed on widespread CCS deployment because of the assumed residual CO_2_ emissions from the energy sector.

More recently both modeling work for open-access solvents MEA and CESAR1, e.g., refs 5,12,13 and a limited number of pilot tests (again with these open-access solvents, e.g., refs 14–21 ) have suggested that operation at or near 100% added CO_2_ capture without excessive solvent regeneration energy is feasible, provided that the plant design has sufficient absorber packing (e.g., a requirement for around 24 m of structured packing is indicated in both modeling and experimental tests on different pilot plants, and for both solvents) and is operated at a high enough desorber pressure (around 2.4 bara, again found in both modeling and pilot tests for both solvents) to achieve a low lean loading without excessive energy requirements. Also, of course, the capture plant must have access to enough steam to operate the desorber at the required capture rate under all operating conditions; this is a practical issue that can be met in different ways if sufficient suitable steam cannot be extracted from existing sources, e.g., by adding a steam ejector if the main steam extraction pressures are too low at part loads.^22^

The desorber pressure is critical because the lean loading below which specific reboiler duty (SRD) values start to increase exponentially, termed the “desorber inflection point”, decreases with increasing pressure. This critical role for desorber pressure has not always been appreciated, with some studies (e.g., refs 13,23 ) investigating the energy or cost sensitivity to capture rate assuming a relatively low, fixed desorber pressure and therefore concluding that the low loadings required in the absorber for high capture rates, e.g., ref 5, would unavoidably incur excessively high SRD values. While higher desorber pressures are associated with higher reboiler temperatures, and therefore a greater risk of thermal degradation, Mullen et al.^24^ have examined how the increase in the thermal degradation rate for MEA might be offset, to a degree, by lower leaner loadings, because the carbamate ion of loaded MEA is the main component that is degrading. Solvent degradation rates were predicted to increase by 24–138% as capture rate was raised from 95% to 100%, although this assessment was based on modeling extrapolations from short-term laboratory tests.

Similar degradation modeling studies considering the effect of high reboiler pressures and temperatures in conventional desorber configurations do not appear to have been published for other solvents. However, based on pilot testing experience with CESAR1, when, intentionally, no solvent reclaiming was used, Moser et al.^25^ noted “*While an elevated desorber temperature* [that requires an elevated desorber pressure] *seems to be an effective measure to increase the capture rate at moderate increase of the specific energy demand of the solvent regeneration and to control the nitrosamine concentration in CESAR1, there is always a trade-off between the positive aspects of an increased desorber temperature (higher CO*_2_ *capture rates, higher CO*_2_ *pressure, control of nitrosamine concentration in the solvent) and negative effects (higher pressure and value of the steam needed for the solvent regeneration, increased formation of volatile and non-volatile degradation products, which must be handled or might form hazardous components in consecutive reactions)*.”

The only way, however, to reliably determine the actual operating limits on desorber pressure, and hence reboiler temperature, is to operate for long periods (i.e., 12–24 months^26^) with full solvent management, which is likely to be continuous thermal reclaiming in the case of MEA,^27,28^ and with a representative flue gas source. Different outcomes can be expected for different applications, solvents, and solvent management methods. This paper is not intended to address this issue of long-term solvent management (and related issues such as emissions to atmosphere and corrosion), although the control approach it presents is expected to be important in long-term plant operation to maintain these high capture rates essentially indefinitely, while at the same time optimizing energy performance, despite the inevitable changes in plant operating conditions, solvent composition, etc., that will occur in practice.

The optimum PCC plant performance for a given set of process parameters is typically determined from an SRD “U-curve” derived from physical tests or process modeling runs, where a number of parameters are kept constant, and the absorber liquid-to-gas (L/G) ratio is varied. Examples of U-curves derived from process modeling for constant capture rate with the heat input adjusted as required at each L/G ratio are shown in Figure 1. Also shown in Figure 1 are the modeled lean and rich loadings and the dimensionless center temperature (DCT), the ratio between the vapor phase temperature drop across the upper bed (of two) in the desorber packing and the total vapor phase temperature drop across all of the desorber packing. The significance of the DCT will be discussed further in Section 3.

**Figure 1:**
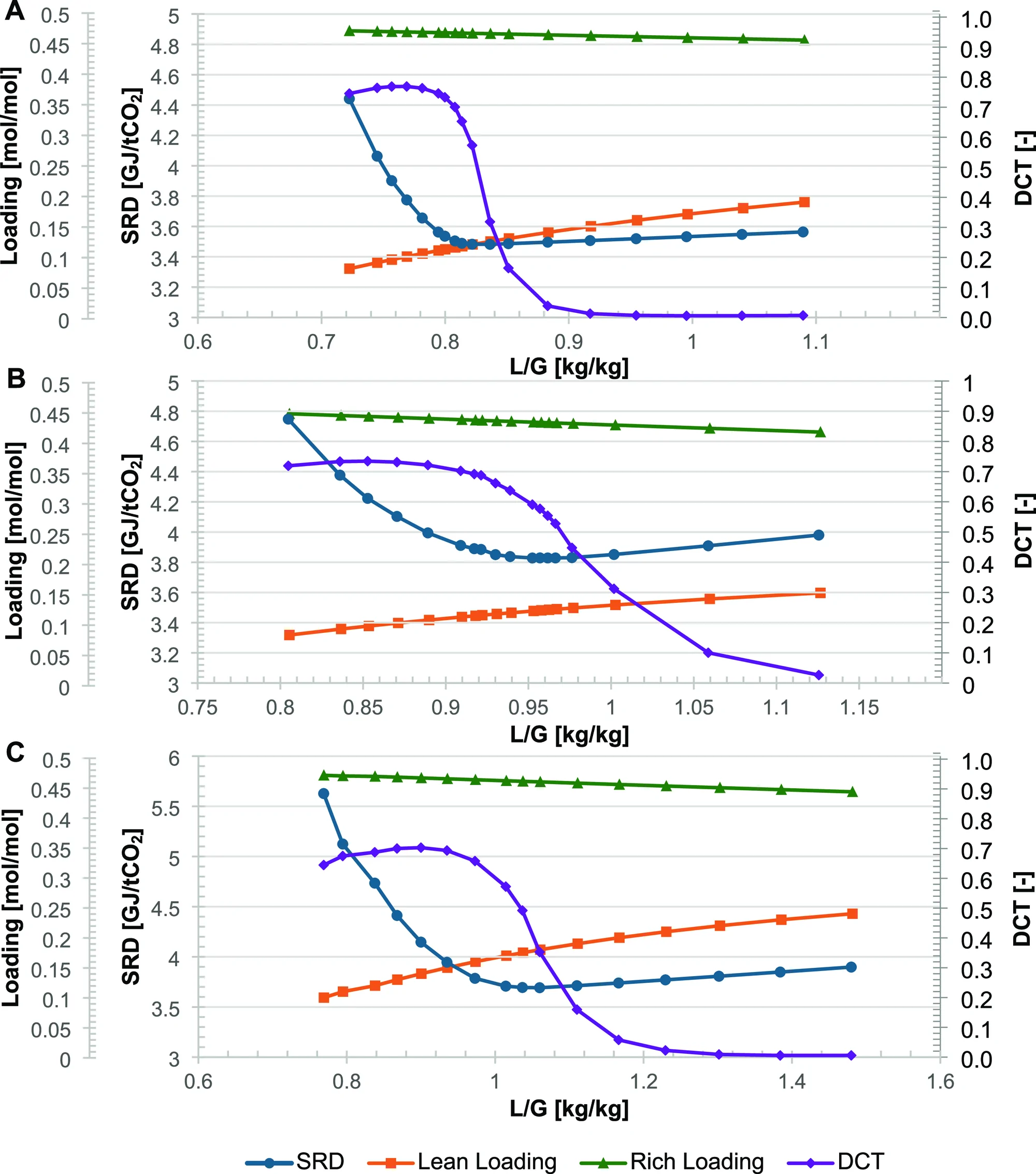
Process modeling examples of SRD U-curves as a function of L/G for a 35% wt MEA solvent: (A) 2.4 bara, 95% fossil-CO_2_ capture; (B) 2.4 bara, 100% fossil-CO_2_ capture; (C) 1.7 bara, 95% fossil-CO_2_capture.

In physical testing, each point on the U-curve is determined by holding conditions as steady as possible, typically for some hours, at what are deemed to be approximately steady-state conditions (e.g., Fosbøl et al.^29^ stated that they used hold times of between 3 and 24 h, out of which 1 to 8 h were used for calculation of average steady-state conditions). The effectiveness of using this method to derive the optimum capture plant operating conditions relies heavily on the capture system inputs, such as flue gas flow, CO_2_ concentration, temperatures for gas and liquid flows, and reboiler steam pressure, remaining approximately constant during the tests. Subsequently applying this practical experience from the U-curve tests in long-term commercial plant operation also requires either operating the commercial capture plant with the same input conditions and control settings as during the tests or, when this is not the case, relying on methods to estimate the adjustments required to the U-curve-optimum plant operating parameters to meet the new conditions.

However, as the relatively narrow bottoms for the U-curves in Figure 1 suggest, PCC plant operating parameters have to be set quite precisely to minimize the SRD, so it may be challenging for operators to estimate new values from either process modeling or past experimental data with sufficient accuracy to accommodate the continual changes that can be expected in practice in the flue gas flow and composition arising from variations in operating conditions, fuel composition and changing ambient conditions plus also the changes in solvent composition as concentration and impurity levels inevitably vary to at least some extent during operation. As noted in trials of Nonlinear Model Predictive Control (NMPC) at Test Centre Mongstad (TCM) “*The main challenge in the optimization problem is localizing minimum reboiler duty. Hitting the U-curve minimum with optimal solvent flow rate is crucial for localizing minimum SRD.* ...*Future work will involve software/model improvement to better fit optimal solvent flow rate to experimental results, especially focused on this range of the U-curve*.”^30^

The alternative to using predetermined operating parameters, from testing or modeling, which this paper will describe, is instead to use continuous feedback control based on generally applicable principles to optimize the desorber performance and then, also using feedback control, to apply the minimum L/G ratio in the absorber that is required to achieve the desired capture rate. This feedback control has been implemented manually in the trial reported here, but it appears that various degrees of automation are also feasible.

Because of its potential importance for ongoing amine PCC deployment, the earliest possible notice has already been given to the CCS community of the likely value of this approach, summarized as STRETCHER—Stripper (i.e., desorber) Tuning for Regeneration Efficiency and Temperature Control for High Exit Rich—in preliminary publications covering the ongoing work.^31–34^ This was done to help ensure that new commercial designs in particular would incorporate the necessary instrumentation; for example, it was not clear that commercial units would install the required thermocouples in the desorber column as a matter of course.

In this full paper on the method, a much-extended modeling exercise is used to explore aspects of the control approach in more detail, and a 75 h pilot test is described that suggests that, as far as control was concerned, a capture rate of around 100% could have been maintained indefinitely, as long as the plant continued to operate satisfactorily.

## 2 Modeling and Experimental Methods

### 2.1 Process Modeling for MEA Performance Trends

The CCSI MEA steady-state model^35^ was used to examine the performance of an amine plant using MEA over a range of operating conditions. The model was implemented by using the built-in e-NRTL thermodynamic framework. The absorber and desorber are rate-based packed columns, both discretized with 100 stages. The absorber is assumed to have three 8 m beds with an intercooler positioned between the two lower beds. Modeling parameters are shown in Table 2.

**Table 2:** Key Assumed Modeling Parameters

parameter	units	value
flue gas from CCGT	kg/s	404
CO_2_ flow rate (inlet)	kg/s	29.76
flue gas CO_2_ concentration	Vol% (dry)	5.27%
absorber packing height	m	24
absorber diameter	m	11.4
absorber packing type	[-]	MellapakPlus 252Y
absorber number of beds	[-]	3
intercooling return temperature	°C	30
intercooling stage height (From bottom of absorber)	%	33
*F* factor	Pa^0.5^	3.62
liquid-to-gas ratio	kg/kg	variable
desorber CO_2_ delivery pressure	Bara	2.4
desorber packing height	m	20
desorber diameter	m	7.4
direct contact cooler exit temperature	°C	40

Other modeling variables are given in figure captions and legends. The flue gas used for the process model is from a GE H-Class 1 × 1 CCGT (GE 9HA.01) (Figure 2).^12^

**Figure 2:**
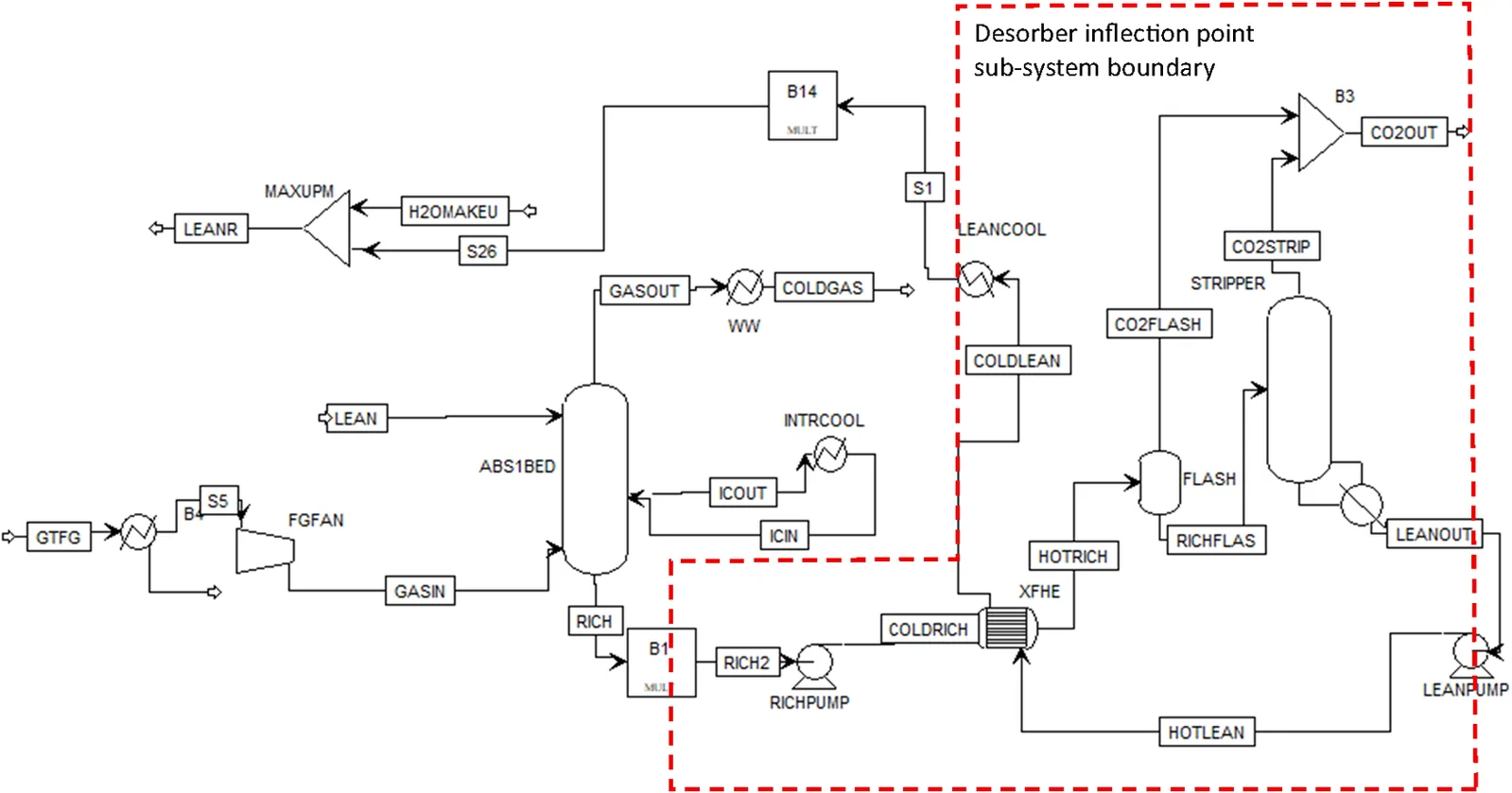
Process flow diagram in Aspen CCSI for integrated steady-state performance modeling (U-curves) and absorber corner calculations with subsystem used for desorber inflection point (red boundary).

### 2.2 Pilot Testing

The pilot facility used for an extended demonstration of the control methods was the Guangdong Carbon Capture Test Platform on the Haifeng Power Plant in China with a capacity (in these tests) of approximately 30 tCO_2_/day. The pilot facility receives flue gas and services from the power plant, an important consideration given that the flue gas CO_2_ content and also steam and cooling water temperatures to some extent are unavoidably affected by the operating patterns of the power plant. This variability does, however, have the benefit of mimicking some of the challenges of other real-world applications, where similar uncontrolled input fluctuations occur. Reboiler steam was obtained from the nominal 10 bar utility steam supply on site, but pressure and temperature varied over time with the main power plant operating conditions, as reported in Section 4.1.

The inlet flue gas CO_2_ concentration was approximately 8–16% v/v dry to the absorber, which has 3 packed beds, each with 5.3 m of packing of Sulzer Mellapak CC structured packing, and a diameter of 1.8 m. Intercooling was used between the two lower beds.

The desorber has two packed beds of Sulzer Mellapak CC for solvent regeneration, each with a height of 4.26 m and a diameter of 1 m. The once-through thermosyphon reboiler uses a shell and tube design. A diagram of the desorber arrangement is given in Figure 3. This shows the location of the DCT measurement thermocouples, in the vapor spaces above and below the packing beds and in the central gap. For further pilot plant details, see refs 36,37 .

**Figure 3:**
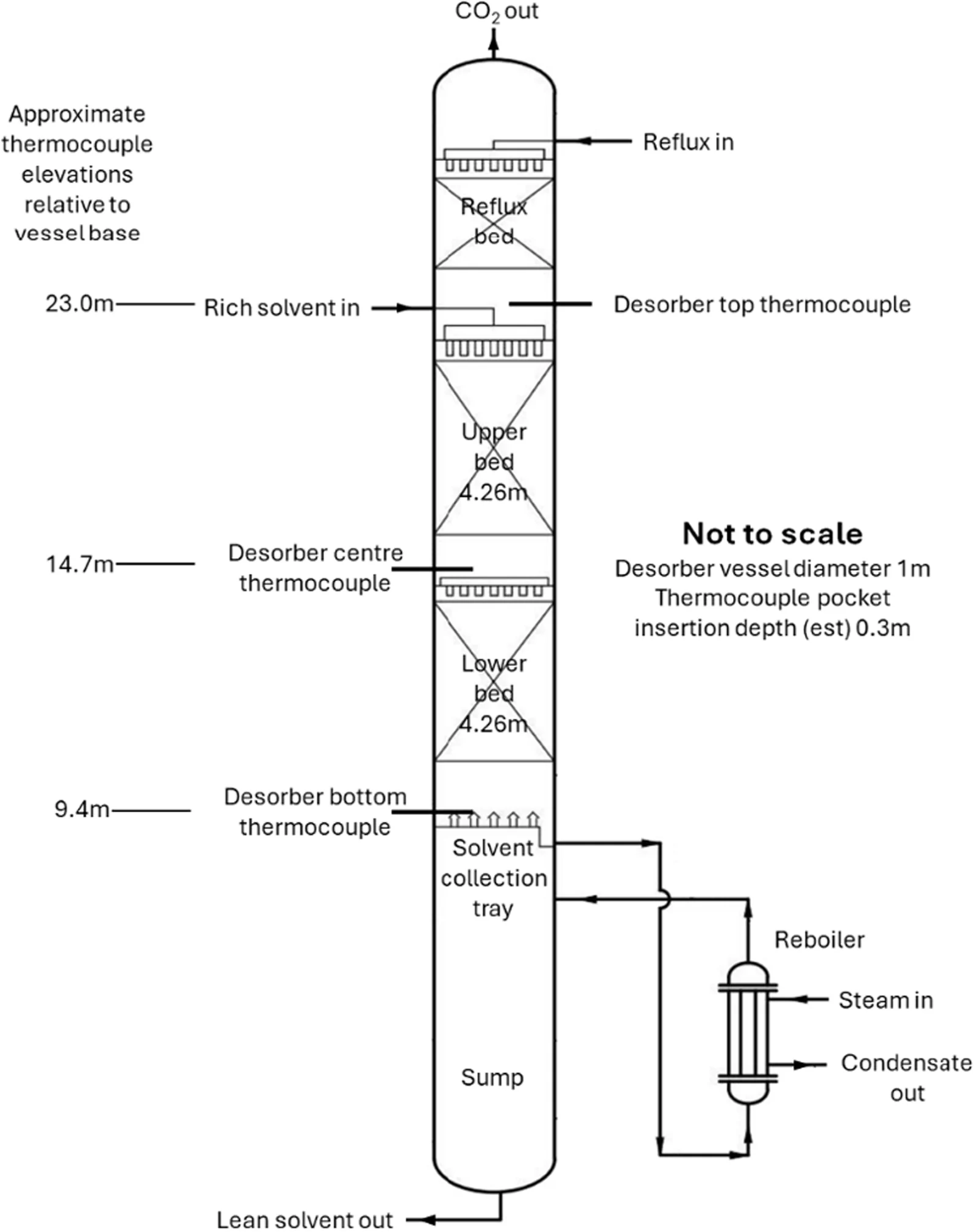
Desorber diagram showing thermocouple location and relative heights.

Note that the key factor in making the temperature between the two packing beds a ‘central’ value is not the axial distances between them but that the packing beds are of equal depth. The interaction between the solvent and the vapor phases can be expected to occur overwhelmingly within the packing, not in the gaps, trays and flow distributors in the desorber.

## 3 Factors Determining the U-Curve Behavior and Implications for Control

The U-curve behavior can be explained by the effect on SRD of variation in the rich loading and the desorber SRD characteristics as the lean loading is reduced. Both are illustrated in the Aspen CCSI modeling results for MEA in Figure 4.

**Figure 4:**
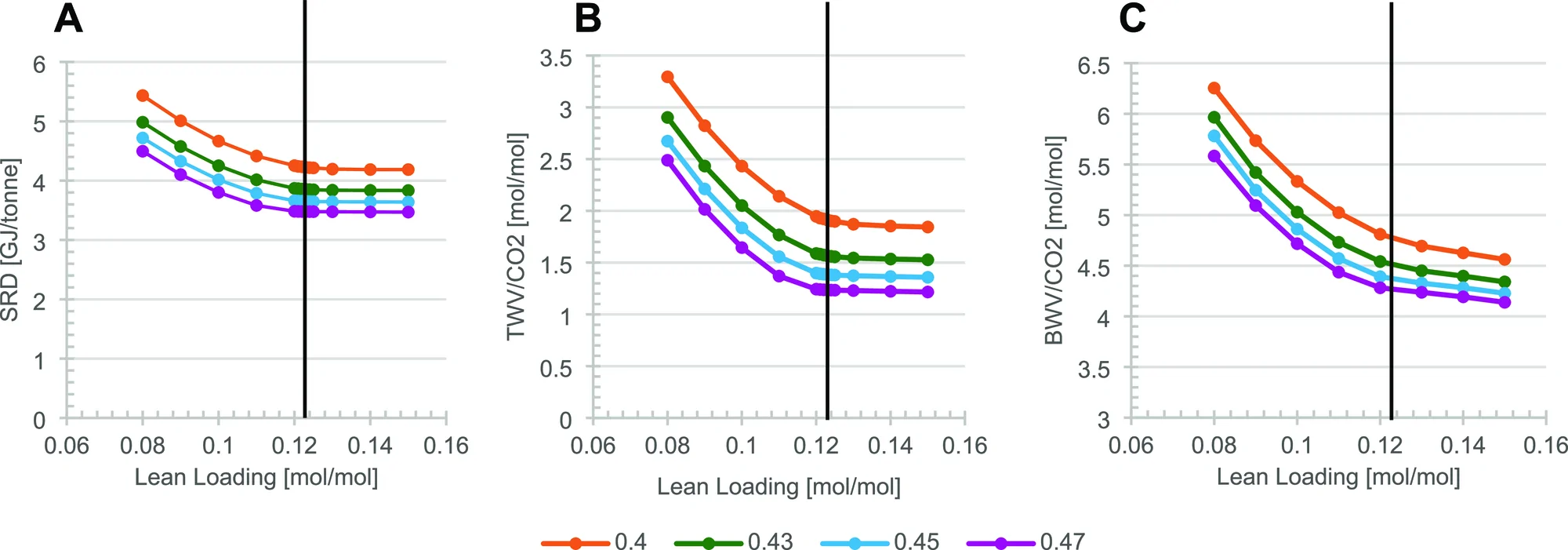
Desorber SRD (A), packing top water vapor (TWV)/CO_2_ mol ratio (B) and bottom water vapor/CO_2_ mol ratio (C) for a range of constant rich loadings and 35% w/w unloaded MEA concentration as a function of lean loading [molCO_2_/molMEA]. Other modeling assumptions are shown in Table 2. The approximate location of the inflection point is highlighted across all the figures.

Note that, in this paper, process modeling is being used to show how suitably applied feedback control can be used to adjust plant controls, using feedback from readily measured parameters, to achieve a target capture rate with near-optimum energy requirements, not to predict what the actual energy requirements, or the associated solvent loadings, etc., would be in practice. As already discussed, exact performance values in physical tests will depend on the state of the solvent and the plant, which will change over time and so cannot be expected to be predicted accurately even if model parameters are fully tuned to match laboratory and pilot plant data on fresh solvents. The qualitative desorber and absorber performance trends presented below can, however, be expected to apply under a wide range of conditions as long as the PCC plant is working reasonably satisfactorily.

### 3.1 Desorber Performance, Rich Loading Effects

The rich loading in the solvent feed to the desorber has a significant effect, with higher values always giving lower SRD values (Figure 4A). The rich loading is not controlled directly by the desorber operating parameters but is influenced indirectly by the lean loading in the feed to the absorber, as will be discussed in Section 3.3.

Lower rich loadings act to increase the SRD partly by requiring a higher solvent flow rate and, hence, more energy to heat the solvent. A lower rich loading also means a higher temperature after the rich solvent flashes as it flows into the desorber, leading to higher levels of water vapor leaving with the CO_2_ at the top of the main desorber packing (top water vapor, TWV, Figure 4B). The higher SRD and higher solvent flow rate is also linked to more heat going into the reboiler and hence more water vapor in total coming out and into the bottom of the packing (bottom water vapor, BWV, Figure 4C), although the local water vapor/CO_2_ ratio in the desorber sump, below the bottom of the packing, is constant at any given lean loading because of vapor liquid equilibrium (VLE) considerations, i.e., the proportion of the total CO_2_ released in the reboiler, rather than in the desorber packing, increases with decreasing rich loading.

### 3.2 Desorber Performance, Lean Loading Effects

The lean loading can be actively controlled by adjusting desorber operating parameters, particularly the steam flow to the reboiler. As the desorber lean loading is reduced at constant rich loading, the SRD is almost constant until a critical condition is reached, termed the desorber inflection point, after which it starts to rise rapidly with further lean loading reductions (Figure 4A). At the inflection point, the amount of water vapor in the flue gas exiting with the CO_2_ at the top of the absorber packing (top water vapor, TWV, Figure 4B) also changes from being nearly constant to rising rapidly. The latent heat of this increasing amount of vapor is the main factor determining the increase in the SRD.

The top water vapor can be viewed as having entered at the bottom of the packing (bottom water vapor, BWV, Figure 4C) after being generated in the reboiler. The amount of water vapor leaving the desorber sump and entering the bottom of the desorber packing rises as the lean loading falls to give the necessary lower CO_2_ partial pressures for the VLE in the desorber sump. At lean loadings down to the inflection point value, however, the potential heat demand for warming the solvent flowing down through the packing and releasing some of its CO_2_ is still greater than the heat available when the vapor phase is cooled to near the temperature of the rich solvent at the top of the packing. As a result, none of this water vapor produced in the reboiler for VLE reasons is “wasted”; all of its usable energy is recovered, with the balance of the overall regeneration energy then being supplied by further direct heat input to the solvent in the reboiler. But, at the desorber inflection point, the heat available from the water vapor entering the packing becomes equal to the heat demand from the downcoming solvent. Then, when more water vapor has to be generated in the reboiler to achieve even lower lean loadings, its available latent heat cannot all be usefully recovered in the packing and so the TWV rises (and this is irrespective of how much desorber packing is available, because it is just a result of an energy imbalance).

Note that this vapor/liquid phase heat balance condition, not any particular lean loading, is the definition of the inflection point, and it is the fundamental reason why an SRD minimum occurs around the inflection point. Although this was not realized when inflection point behavior was initially described,^5^ the transition from insufficient to excess heat availability from the bottom water vapor entering the packing can readily be observed in desorber temperature profiles, as shown in Figure 5. When the heat available from the BWV is lower than that which the liquid phase can absorb, the temperature is close to the (relatively low) value at the top across much of the packing. When the heat available in the BWV is greater than the heat demand from the liquid phase, the temperatures across much of the packing are close to the (relatively high) value at the bottom of the packing. Only at the inflection point is there a transition state where temperatures vary approximately uniformly along the packing direction.

**Figure 5:**
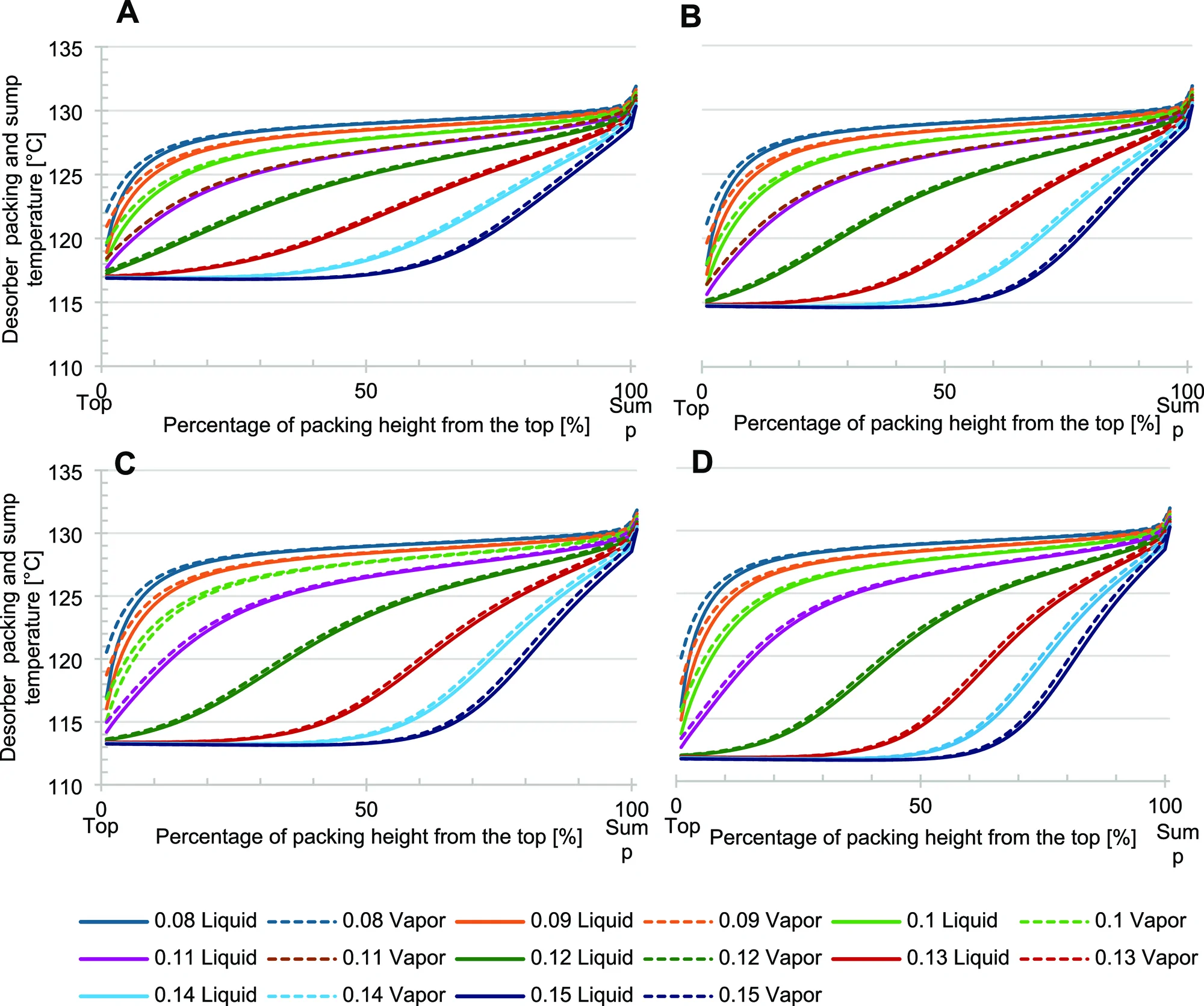
Desorber column temperature profiles of various lean loadings at a rich loading of 0.4 (A), 0.43 (B), 0.45 (C), and 0.47 (D) molCO_2_/molMEA from modeling. The calculated vapor and liquid temperatures are shown with dotted and continuous lines, respectively.

The liquid and vapor phase temperatures are generally predicted to be close in this modeling study, except where there are fundamental thermodynamic reasons why they cannot be. One example of this is that, in all cases, the temperature of the vapor entering at the bottom of the packing, which is close to being in equilibrium with the lean solvent, is higher than the temperature of the liquid leaving the packing, which will always be at a slightly higher loading than the lean value in the sump due to the heat input in the reboiler. The other example is when the BWV is above the inflection point value and the vapor phase cannot be cooled to close to the liquid phase temperature at the top of the desorber packing due to the heat demand/supply imbalance discussed above.

This pattern of variation in desorber temperature profile with lean loading and SRD was initially confirmed experimentally by pilot plant measurements from the National Carbon Capture Center (NCCC),^38^ undertaken as part of unpublished MEA baseline testing conducted in 2022, presented in Figure 6. These observed desorber column temperature profiles (the sump and top temperatures are expected to be the vapor phase values, but it is not possible to tell whether the reading is the vapor or the liquid phase at other points) for approximately steady-state operating points with 30% MEA at 1.7 bara show a similar transition as the loadings passed from low to high values; this was also observed to be at the point where SRD values started to increase with decreasing lean loading, when allowance was made for variations in rich loading.

**Figure 6:**
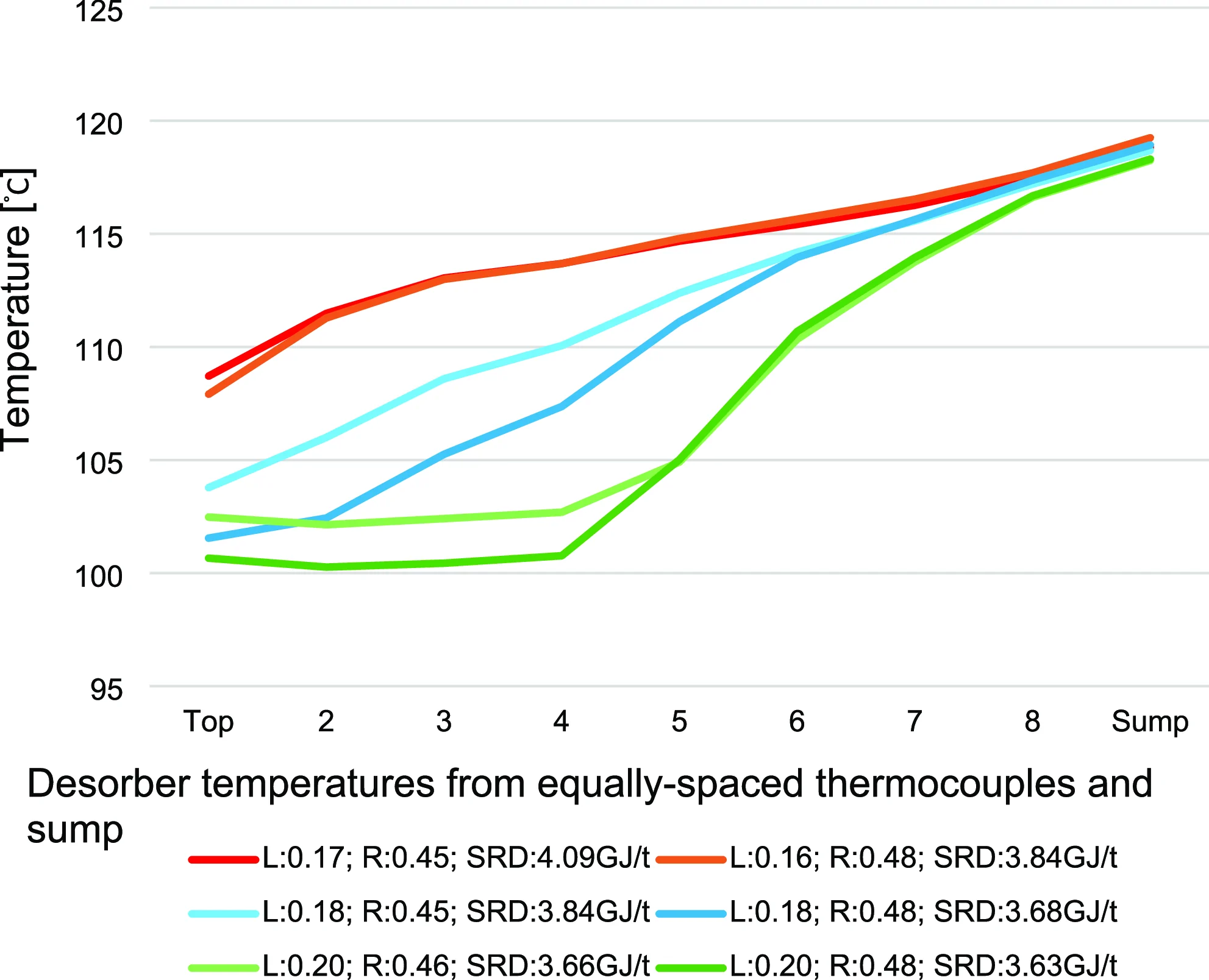
NCCC steady-state tests (using 30% w/w MEA and with a desorber pressure of 1.7 bara) show desorber temperature profile changes in passing through the inflection point behavior (legend: lean loading, rich loading, SRD).

It is therefore clear that the desorber temperature profile can be a useful indicator of when the inflection point is reached, as it inherently reflects the underlying thermodynamics of why the inflection point is occurring, i.e., water vapor ‘breakthrough’ in the desorber packing. Absolute desorber column temperature values are not a reliable guide to inflection point behavior, however, as desorber column temperatures also vary with rich loading, desorber pressure, and solvent amine concentration. Instead, we have used a dimensionless center temperature (DCT), defined as the following ratio for the vapor phase temperatures

1 $$DCT=\frac{T_{centre}-T_{top}}{T_{bottom}-T_{top}}$$

In typical desorber arrangements with two packing beds, it is convenient to measure all three temperatures explicitly in the vapor phase, in the spaces below, between and above the beds, as shown in Figure 2. Moreover, these temperatures do not need to be measured with very high accuracy, or a very low noise level, in the readings, since the change in the center temperature is of the order of 15 K (see Section 4.1 for a more detailed discussion of errors and signal noise).

There is also a question of the extent to which, in large commercial plants, conditions will not be uniform across an amine desorber cross section, but no public domain information on this has been identified. It may then be possible to use DCT readings from various points across the desorber cross section to identify non-uniformities and perhaps also to correct them by modifying the solvent flow or heat supply to individual reboilers, if that is the cause of the problem. Foaming in the desorber or damage or fouling of the internal parts might also lead to non-uniform or misleading DCT values, although these problems will be minimized as far as possible by operators.

As the desorber goes through the inflection point, the DCT inherently transitions from a value below 0.5 to a value above 0.5. DCT values in Figure 1 suggest that the U-curve minimum occurs at a DCT value close to 0.5 for MEA under the conditions examined. Also, for practical control purposes, it is important to have a target DCT value that is not too close to either extreme because, near the upper and lower flat regions in the DCT curve, DCT changes with reboiler heat input (or other factors that affect inflection point behavior) will not be so apparent. As a result, a target DCT value of around 0.5 may be a reasonable initial choice. In practice, though, plant operators can be expected to build up experience on precise DCT values to control to, with these values possibly also varied for different operating circumstances (e.g., part load vs full load) and different market conditions (e.g., electricity price). For example, as discussed in the Appendix, lower DCT values appear to give the optimum results for the very low rich loadings that might be encountered in amine DAC absorbers.

Figure 7 shows how DCT values vary with lean loading for a range of rich loadings (0.4, 0.43, 0.45, and 0.47) and rich concentrations (33, 35, and 37% w/w MEA). It can be seen that, even with this range of rich compositions, controlling the desorber to a constant DCT value, e.g., 0.5, results in a very narrow range of lean loadings being produced, with only a limited extension of the lean loading range if a DCT range of, e.g., 0.3 to 0.6 is able to be achieved in practice; this is relevant for the discussion of absorber control in the next section.

**Figure 7:**
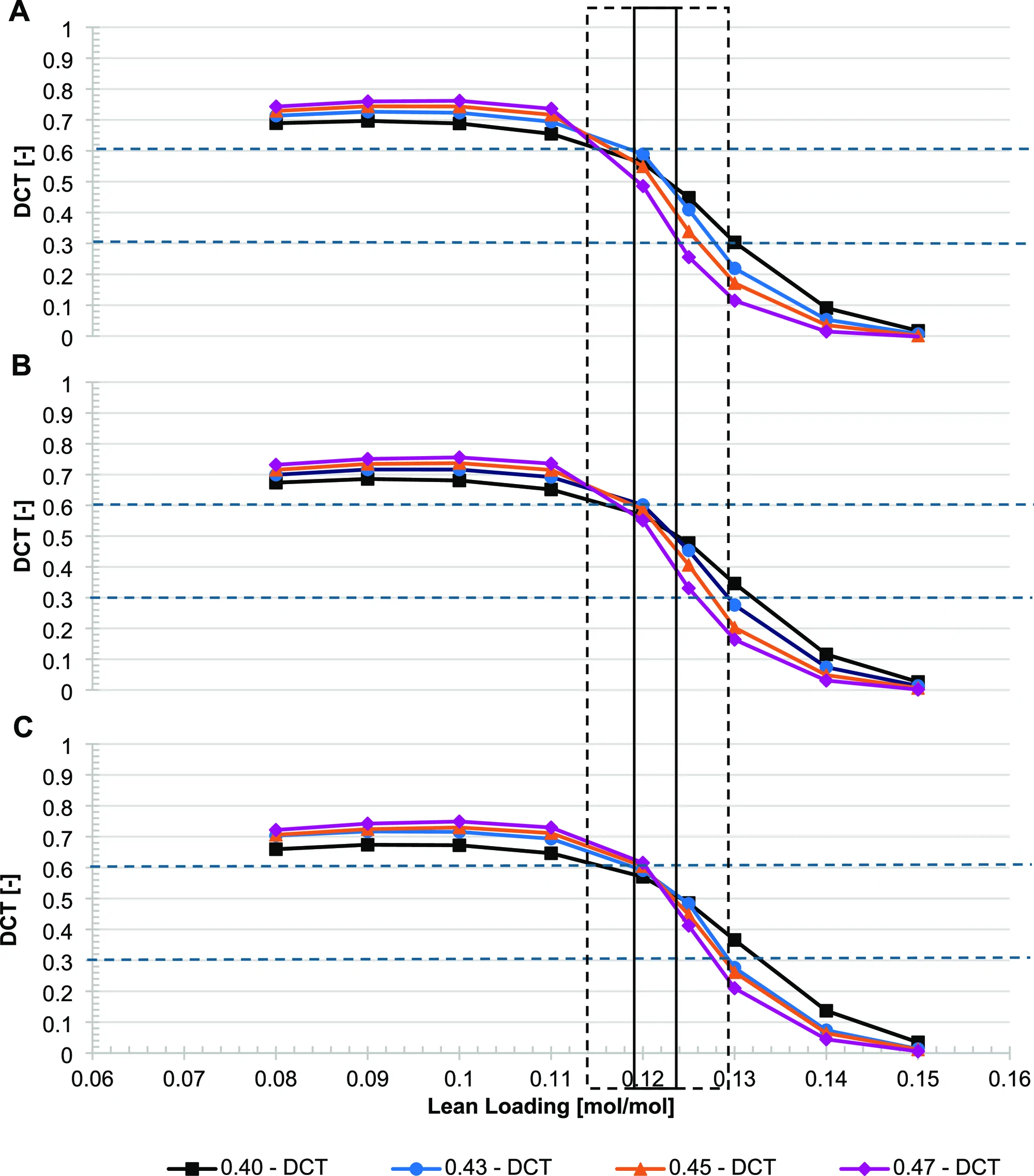
Dimensionless center temperature values at (A) 33, (B) 35, and (C) 37% w/w MEA. The solid vertical lines are the limits of lean loading for a dimensionless center temperature value of 0.5 at the different MEA concentrations, with the narrow range illustrating the relative sensitivity to operational changes in solvent concentration. The dotted vertical lines show the wider, but still acceptable lean loading range for subsequent absorber control purposes, for DCT values from 0.6 to 0.3 across the range of rich solvent loadings and concentrations. Possible upper and lower DCT control limits are shown by the dotted horizontal lines.

A limit on using the DCT value as a guide to whether or not an unusable excess of water vapor is being produced in the reboiler is how fast conditions in the reboiler and desorber sump, and consequently the amounts of CO_2_ and water vapor entering the desorber column, respond to changes in the heating steam flow to the reboiler. As will be discussed in detail later, virtually instantaneous (<2 min delay) reboiler/sump temperature responses to changes in steam flow were observed with the once-through thermosyphon reboiler in the test data reported. Some different reboiler types are shown in Figure 8; the rapid response of once-through thermosyphon reboilers is achieved because they take the full solvent flow exiting the bottom of the desorber packing, heat it, and flash the mixture into the sump to produce the final lean solvent. This type of reboiler also appears to be universally used in full-scale plants.^10,39–41^ Once-through thermosyphon reboilers may, however, be subject to cyclical flow fluctuations, also known colloquially as “chugging”,^42^ although, as will be described later, some amount of this occurred in the plant trial reported here and the DCT control method still performed well over this period and with the overall performance similar to the rest of the test period when the reboiler was more stable.

**Figure 8:**
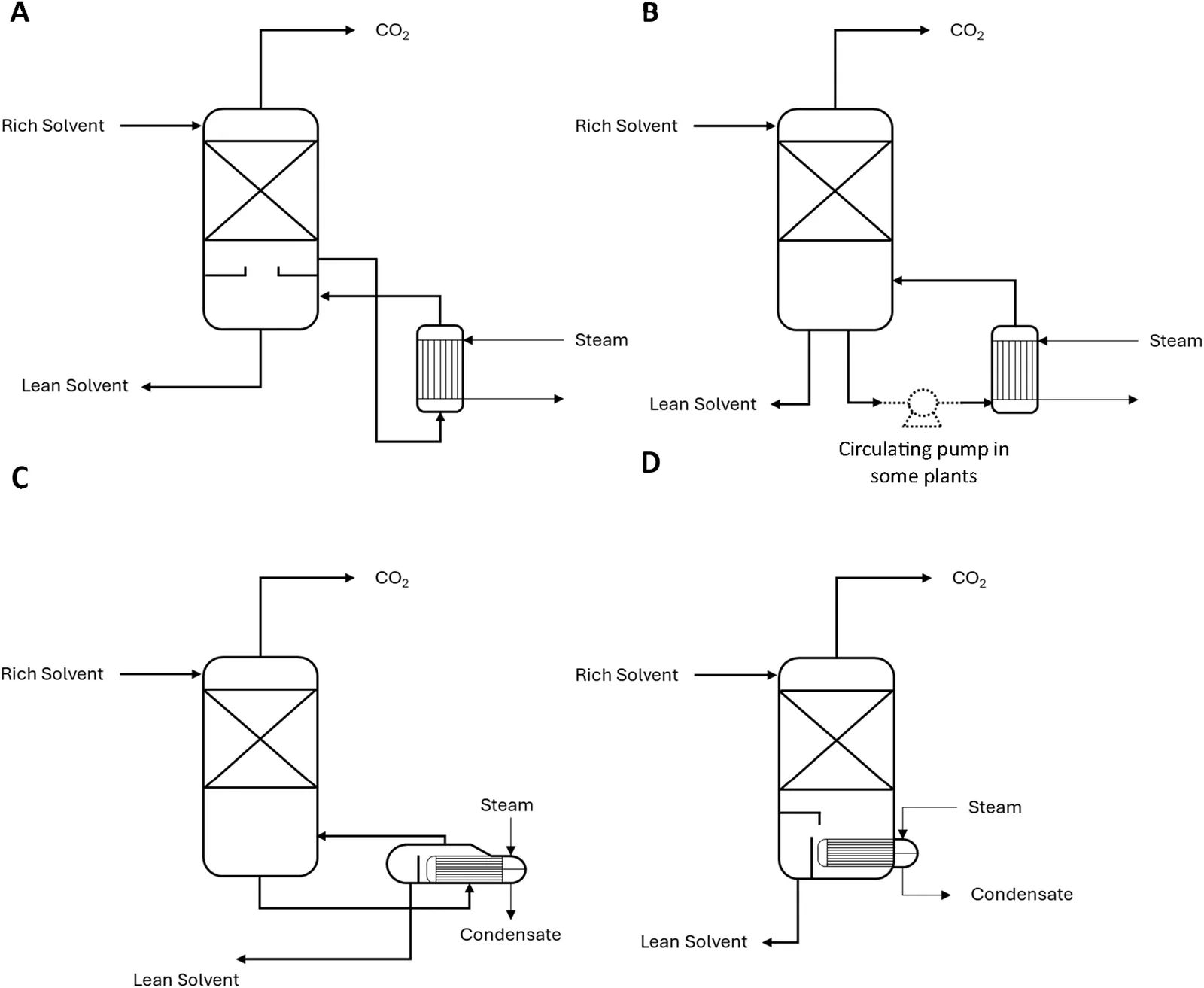
Once-through thermosyphon and other reboilers—diagrammatic arrangement (reflux arrangements at the top of the desorber, integrated thermal reclaimers etc. have been omitted for simplicity) (A) Once-through thermosyphon; (B) Natural or pumped circulation thermosyphon; (C) Natural or pumped circulation thermosyphon with external kettle reboiler; (D) Integrated kettle reboiler.

In this context, it is worth noting that a number of pilot plants, including Test Centre Mongstad with a capacity of around 230 tCO_2_/day,^18^ the NCCC with a capacity of around 10 tCO_2_/day,^38^ and the Energy Innovation Centre (EIC) at the University of Sheffield with a capacity of about 1 tCO_2_/day,^43^ employ variations on circulating reboilers. In these systems, the CO_2_ loading in a relatively large captive solvent volume within the sump/kettle reboiler changes more gradually as the reboiler heat input, and hence CO_2_ and vapor output, is adjusted. Desorber conditions may therefore take on the order of an hour to stabilize^44^ following any changes, compared to minutes for a once-through thermosyphon design.

A further caveat in the application of DCT control is that the desorber packing height must be sufficient for the liquid and vapor phases to approach equilibrium if this is thermodynamically possible. It appears that most pilot-scale plants and full-scale desorbers have sufficient packing for this to be the case. However, in pilot units with relatively short packing heights (e.g., around 5 m or less), this deficiency may not immediately be obvious during testing because the desorber will still successfully operate to produce lean amine. While the SRD will be perceived as having a high value as a result, this may be assumed to be due to other factors, such as operating at a lean loading below the inflection point value, since CO_2_ temperatures at the top of the desorber packing will be higher than incoming rich solvent temperatures, or due to the higher heat losses typical of smaller plants. Conversely, having excess desorber packing will make a minimal impact; as the vapor and liquid phases approach equilibrium, extra packing can make relatively little difference to absolute values.

Whilst the principle of DCT control in the desorber is modeled here using MEA, the desorber operation method can be expected to be applicable to other solvents that use water vapor stripping in the desorber. For example, in a recent study by Moser et al.^21^ conducting a series of systematic test runs using CESAR1, it was stated that, for given parameters of CO_2_ capture efficiency, desorption temperature, and height of packing, the optimal SRD was achieved at solvent flow rates that gave a “*linear temperature profile*” in the desorber. Similarly, Løge et al.^45^ used CESAR1 and demonstrated that the lowest SRD was achieved when the DCT was near 0.5 when modifying the proportion of solvent that bypasses the cross-flow heat exchanger. Tobiesen et al.^46^ also shared a detailed desorber temperature profile (for their trial number 97), representing a capture ratio of over 95% with a DCT of around 0.5 and a lower SRD compared to other runs around the same capture ratio. Another study using blends of AMP and piperazine reported desorber temperature profiles for various lean loadings at the Kaiserslautern Pilot Plant.^47^ This study demonstrated that, at moderate lean loadings, the temperature profile develops along the entire length of the desorption column. Conversely, at lower lean loadings, below the inflection point value, the profile remained relatively flat until close to the top of the desorption column, whereas at higher lean loadings, desorption occurs mainly in the reboiler. Both of these extreme cases correspond to DCTs that are either much lower or much higher than 0.5.

### 3.3 Absorber Operation and Control

With a supply of lean solvent available at near-constant loading, feedback control of the absorber might appear relatively straightforward; simply adjust the L/G ratio until the required outlet CO_2_ level is reached. However, there are some complications in doing this at high capture rates because of the very precise L/G ratio matching required to achieve an optimum rich loading.

Modeled steady-state absorber performance curves as a function of absorber L/G ratio are shown in Figure 9. These show a characteristic ‘absorber corner’ behavior in the region of 100% of added CO_2_ capture. Beyond the ‘corner’ value, increasing the solvent flow rate results in relatively small changes in exit CO_2_ concentration but relatively large changes in rich loading and hence SRD. This contrasts with the trends at lower capture levels, where exit CO_2_ changes rapidly with solvent flow (and so L/G) and the rich loading is relatively constant, limited by the VLE with the incoming flue gas. Consequently, if the exit flue gas CO_2_ analyzer has limited resolution at very low CO_2_ levels, increases in liquid flow beyond the required value might not be readily evident from the measured exit CO_2_ values. Additional problems with using the exit CO_2_ measurement for absorber control could also arise from the time lag before a change in absorber inputs is reflected in the exit CO_2_ level; this is discussed further in Section 4.

**Figure 9:**
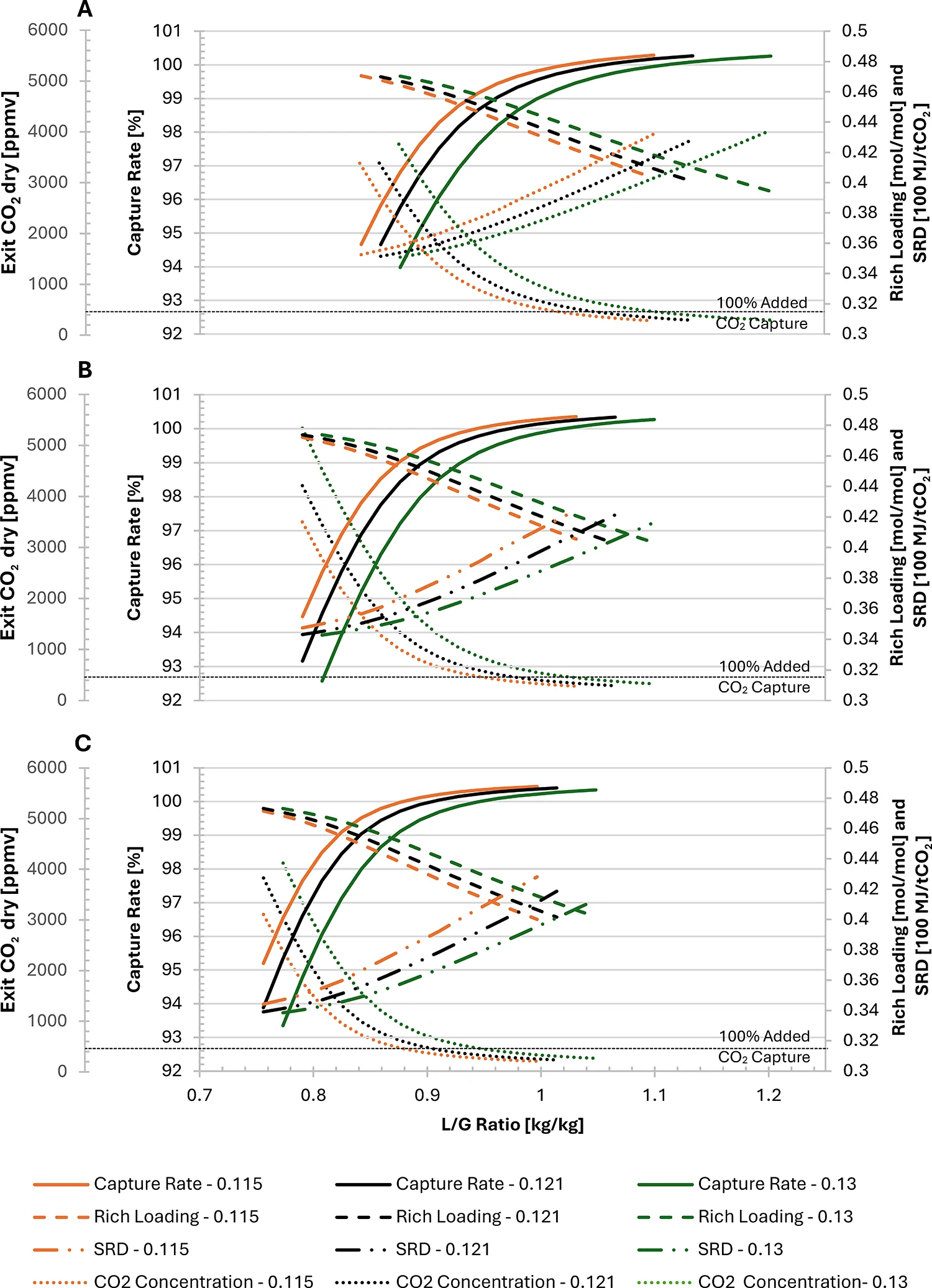
Illustrative absorber corner graphs for three solvent concentrations and three lean loadings: (A) 33 wt %, (B) 35 wt %, and (C) 37 wt % MEA concentrations (unloaded). Lean loadings of 0.115, 0.121, and 0.13 are shown in orange, black, and green, respectively. Capture rate is expressed as the fraction of added CO_2_ in the flue gas. The horizontal black dotted line represents the outlet CO_2_ dry concentration corresponding to 100% added CO_2_ capture.

Figure 9 also shows the link between lean and rich loadings for a given capture rate and the importance of having a low enough lean loading if high capture rates are to be achieved with moderate SRD values. This link might not be immediately apparent because, in all cases, a higher lean loading (0.13 in this example) gives a higher rich loading and, hence, lower SRD at a given L/G ratio. However, this is at a significantly lower capture rate, and so, to achieve the same capture rate with a higher lean loading, a higher L/G ratio is required. Under these conditions, the rich loading is lower than for a lower lean loading at the same capture rate, and the SRD is therefore higher.

## 4 Demonstration of Control Methods in Pilot Plant Testing and Observed Plant Response Times

Testing, principally of the DCT method of desorber control, has been carried out successfully at the Guangdong Carbon Capture Test Platform on the Haifeng Power Plant in China (see Section 2 for pilot plant details) at around 30 tCO_2_/day capture rate, initially in 2024 for periods of approximately 6 h^37^ and in 2025 for a continuous run of 75 h using approximately 35% w/w MEA and a desorber exit pressure setting of 2.4 bara.

The main perturbation in the capture plant inputs was the CO_2_ concentration in the flue gas coming from the power plant stack, which varied with the power plant load and other operating factors. The pilot plant was not designed for 100% capture and, in particular, has only 16 m of absorber packing.^36,37^ To achieve high capture rates with reasonably high rich loadings, the absorber was therefore lightly loaded, with the lean solvent flow constrained to the minimum value expected to be feasible with the installed liquid distribution system. Then, rather than keeping the flue gas flow constant and varying the solvent flow to maintain an appropriate L/G ratio, the flue gas flow had to be adjusted to achieve the desired absorber exit CO_2_ level for approximately 100% added CO_2_ capture. This was achieved using readings from the lower-accuracy and lower-precision (in the ppmv CO_2_ range) plant gas analyzer that was connected to the operators’ logging screens, cross referencing against readings relayed to the control room from a temporarily installed, higher precision ppmv gas analyzer (Senseair K30 NDIR analyzer, accuracy stated +/–30 ppmv +/–3% of reading) on the exit from the plant analyzer.

In most applications, the flue gas flow as well as its composition will be set by the plant to which it is attached, and the required L/G ratio will have to be achieved by adjusting the lean flow rate. Consequently, while these tests demonstrate what can be achieved on capture rate and SRD when an appropriate absorber L/G ratio is maintained, further testing is required to explore absorber control behavior. These tests did, however, provide useful information on likely absorber exit CO_2_ and rich loading response times that suggest improved methods for absorber control; these will be discussed in more detail below.

### 4.1 Desorber DCT Control

As described above, the Haifeng pilot plant uses a shell-and-tube once-through reboiler, and the desorber has two beds of packing with thermocouples below and above the packing and a further one in between the beds for the center temperature measurement. Figures 10 and 11 show how operators found it entirely feasible to use the existing control system to manually modulate the reboiler steam flow control valve so as to keep the center temperature close to midway between the bottom and top temperatures as externally applied conditions for the capture plant, principally the flue gas CO_2_ content and also the reboiler steam supply pressure, changed due to the commercial operation of the coal power plant to which it is attached.

**Figure 10:**
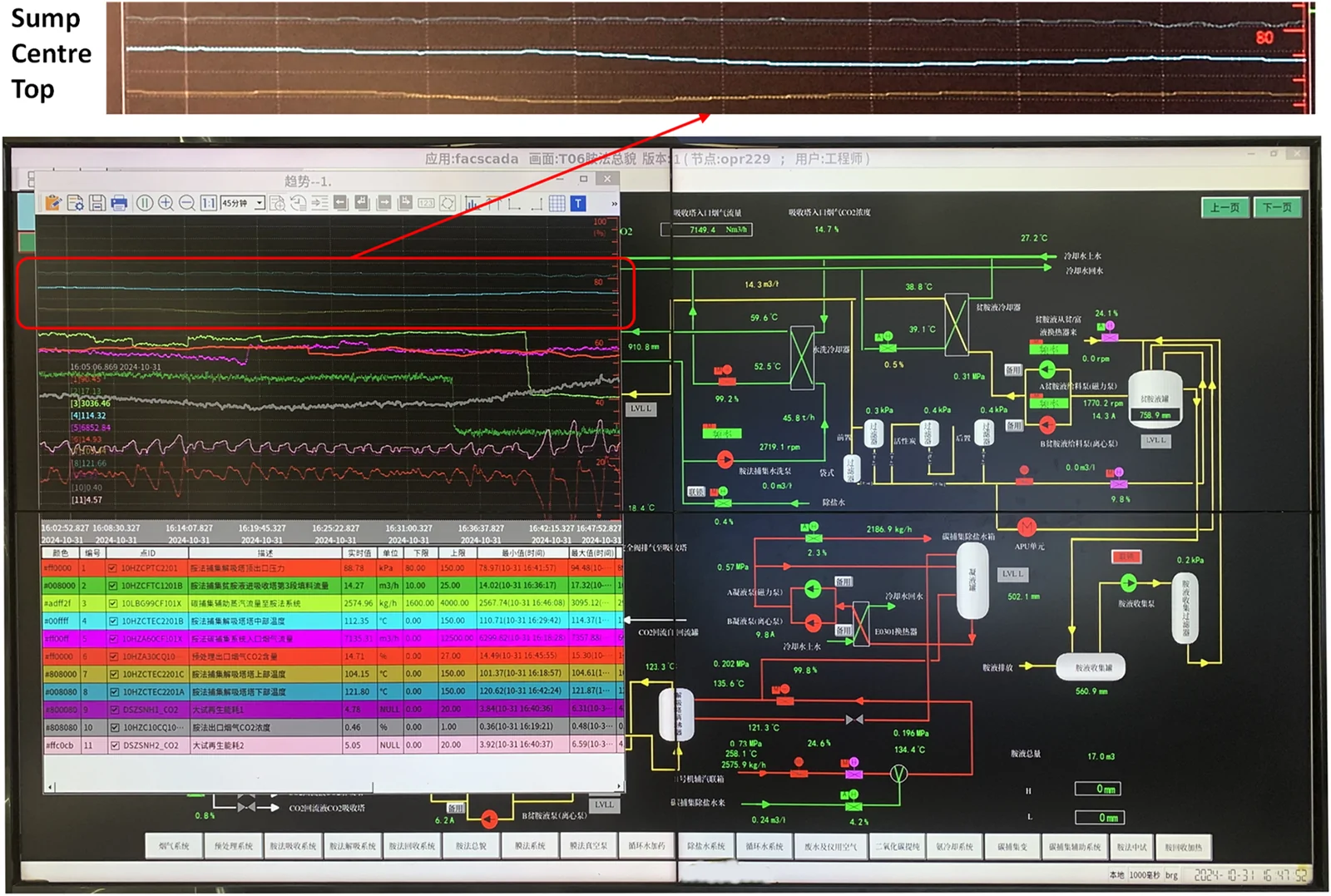
Pilot plant control screen of the Guangdong Carbon Capture Test Platform showing dimensionless center temperature control. The zoomed-in area circled in red in the bottom picture and shown in the top picture is a plot of the desorber bottom, center, and top temperatures over a period of approximately 1 h.

**Figure 11:**
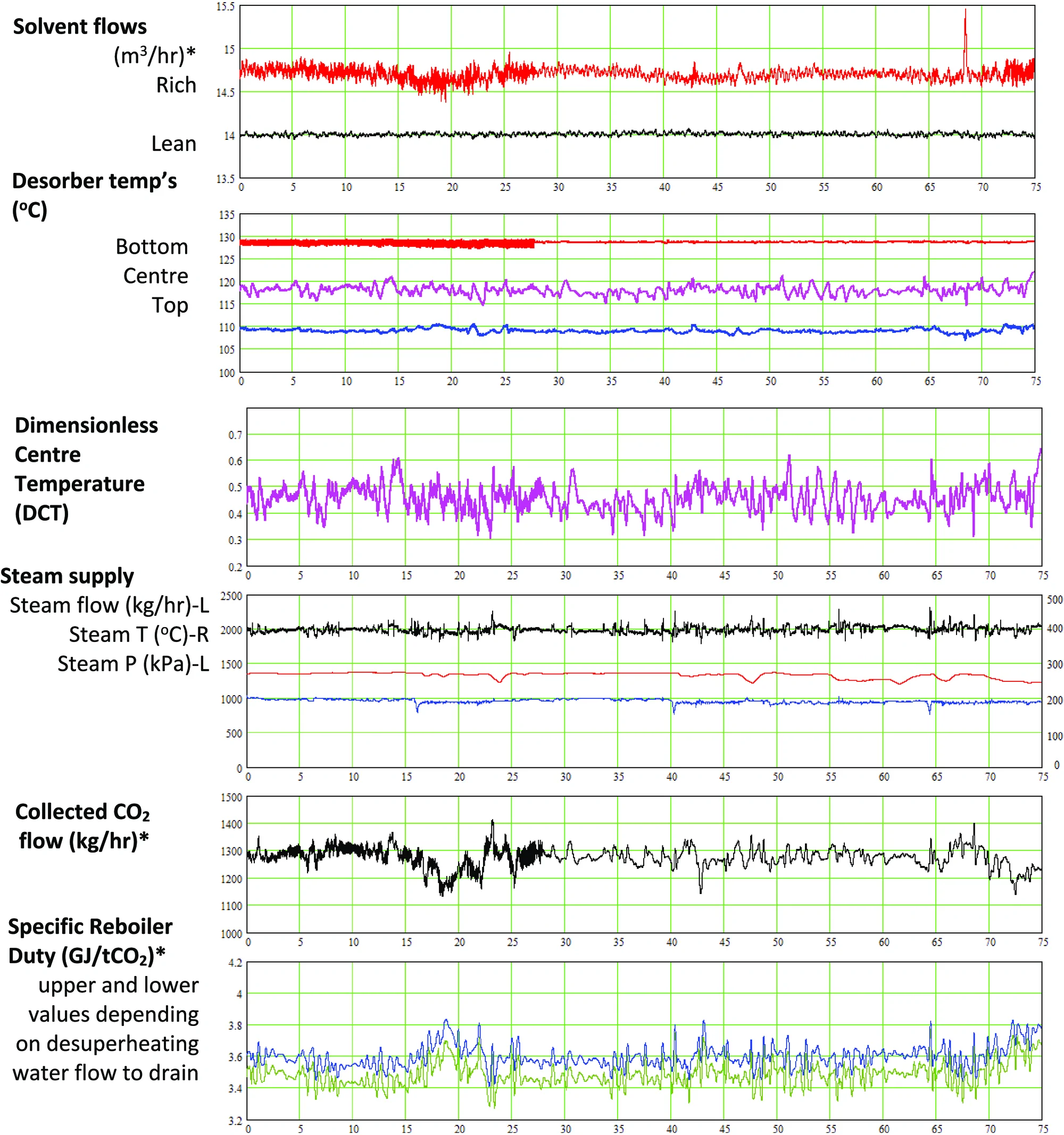
Desorber parameters over a 75 h period of active control (*30 min rolling averages).

In these tests, rich solvent flows to the desorber were set by the absorber sump level controller, resulting in slightly more variation in the rich flow to the desorber than in the lean flow to the absorber, as shown in Figure 11. No provision was available to adjust the level controller to even out the rich flow, which would probably have been advantageous. The lean flow out of the desorber (unrecorded) was also set by level control in the desorber sump, and desorber pressure was controlled automatically at the set value of 1.4 barg (∼2.4 bara). The principal desorber control action by the operators was to adjust the position of the medium-pressure steam valve supplying the reboiler to keep the desorber packing center temperature midway between the bottom (packing inlet) and top (packing exit) temperatures, or slightly lower (i.e., a DCT of 0.5 or slightly lower), using visual judgment (Figure 10). This was generally successful, as shown in Figure 11, with an average DCT value of 0.456 and standard deviation of 0.051, and estimated SRD values, based on the amount of CO_2_ released in the desorber, were entirely acceptable with an average between 3.49 ± 0.01 and 3.60 ± 0.01 GJ/tCO_2_, depending on the (unknown) temperature of excess spray water drained from the steam line after the desuperheater, upstream of the reboiler.

This successful manual feedback control can be explained by the observed insensitivity of the DCT value to the cyclical reboiler fluctuations that occurred until around hour 29 of the test period, coupled with the relatively short response time of around 8.5 min between steam flow adjustments and DCT changes in this plant (Figure 12a,b), attributed to the use of a once-through thermosyphon.

**Figure 12:**
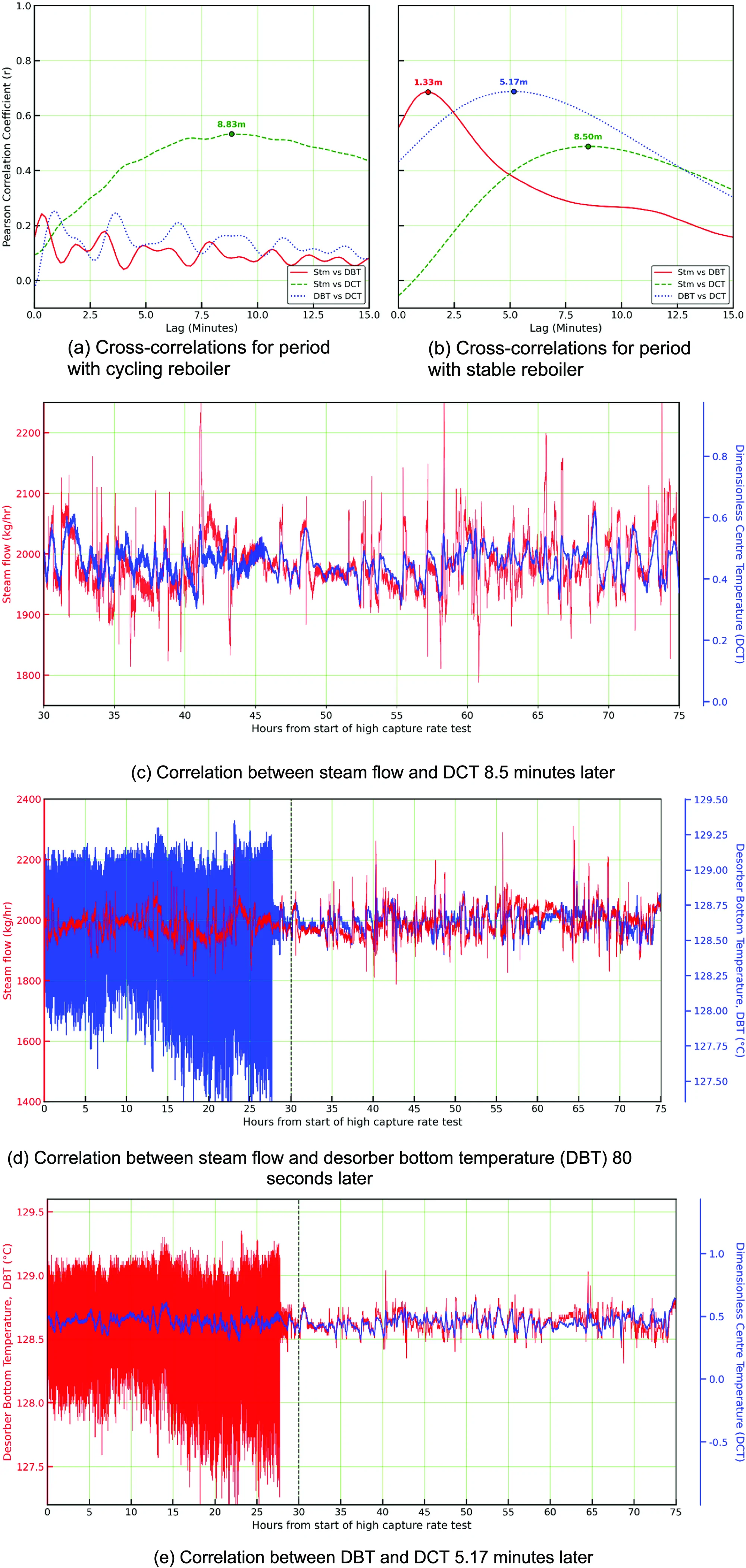
Cross-correlations between reboiler steam flow and DCT and DBT, and between DBT and DCT.

Desorber temperature measurements were undertaken with Class A sensors with a maximum permissible absolute error of ±0.15 K + 0.2% of the reading, so up to ∼± 0.5 K in this case, but, although equipment specification values were not available, the repeatability of the temperature measurements can be seen to be much better (e.g., by inspection of Figure 12e). Therefore, although temperature measurement errors of this magnitude may result in systematic offset of around ±10% in an observed DCT value of 0.5, by reference to Figure 1 this will result in only marginal differences in the achieved SRD and, as discussed in Section 3.2, the precise optimum DCT value to aim for on a particular plant and under particular operating conditions is likely to be refined by operators based on experience over time.

The time shift between reboiler steam adjustments and DCT changes (and the other time shifts described below) was estimated using cross-correlation techniques,^48^ with the most accurate time-shift being taken as the value giving the highest Pearson’s correlation coefficient between an unshifted and a shifted set of data values. Traces for the delayed data set were then calculated using coefficients for the corresponding best-fitting linear regression between the two data sets to allow plotting of the two data series for visual confirmation of the alignment, as shown in Figure 12c–12e.

A signal with an even shorter response time than the DCT would, obviously, offer further advantages for any future feedback control of the steam supply, especially automated control. It was observed that, when the reboiler was operating stably, the desorber bottom temperature (DBT) also followed changes in the steam flow, with a shorter lag of approximately 80 s (Figure 12b,d), and also that the DBT was a good predictor for DCT trends approximately 5.2 min later (Figure 12b,e). It is therefore suggested that feedback control of the desorber steam supply using a target value for the DBT could be considered in future applications. But to do this, two challenges have to be overcome. The first is the need to have stable reboiler operation; this is clearly feasible and desirable but will require attention to details of plant design and operation that are beyond the scope of this paper. The second challenge is that the DBT will be related to the lean loading for a given amine concentration and desorber sump pressure through the vapor liquid equilibrium relationship but, even if all of these factors are known with sufficient precision (and note the expected ∼± 0.5K uncertainty in the absolute value of the DBT in this case), the lean loading does not *per se* indicate whether or not the desorber is operating at the inflection point. The set point DBT value for the feedback controller can, however, be updated as frequently as necessary to take these factors into account, particularly any concentration or other solvent composition changes, by reference to the DCT value achieved. This correction would also overcome any systematic offset in the DBT value, provided that the temperature sensor has adequate repeatability.

### 4.2 Absorber Control Observations and Implications for Possible Improvements

Selected absorber performance parameters are given in Figure 13. With a likely near-constant lean loading due to desorber control, the lean solvent flow rate can be seen to vary inversely to the incoming flue gas CO_2_ content, which exhibits some significant drops during periods of low power plant load, as the operators attempt to maintain a stable exit CO_2_ level of around 500 ppmv. Using this control approach, an exit CO_2_ average of 529 ppmv dry with a standard deviation of 38 ppmv was achieved and, as already noted, SRD values were moderate, which implies satisfactorily high rich loadings were being achieved. This level of control can be contrasted to reported flue gas exit values of an average 436 ppmv, standard deviation 182 ppmv, over a period of 193 h and average 539 ppmv, standard deviation 332 ppmv, over a period of 292 h in two high capture rate tests at RWE’s Niederaussem pilot plant.^21^ These exit CO_2_ changes at Niederaussem imply some changes in SRD as a result of rich loading changes, but no details are given of these, nor of any absorber L/G ratio control methods applied to keep at a particular target exit CO_2_ value.

**Figure 13:**
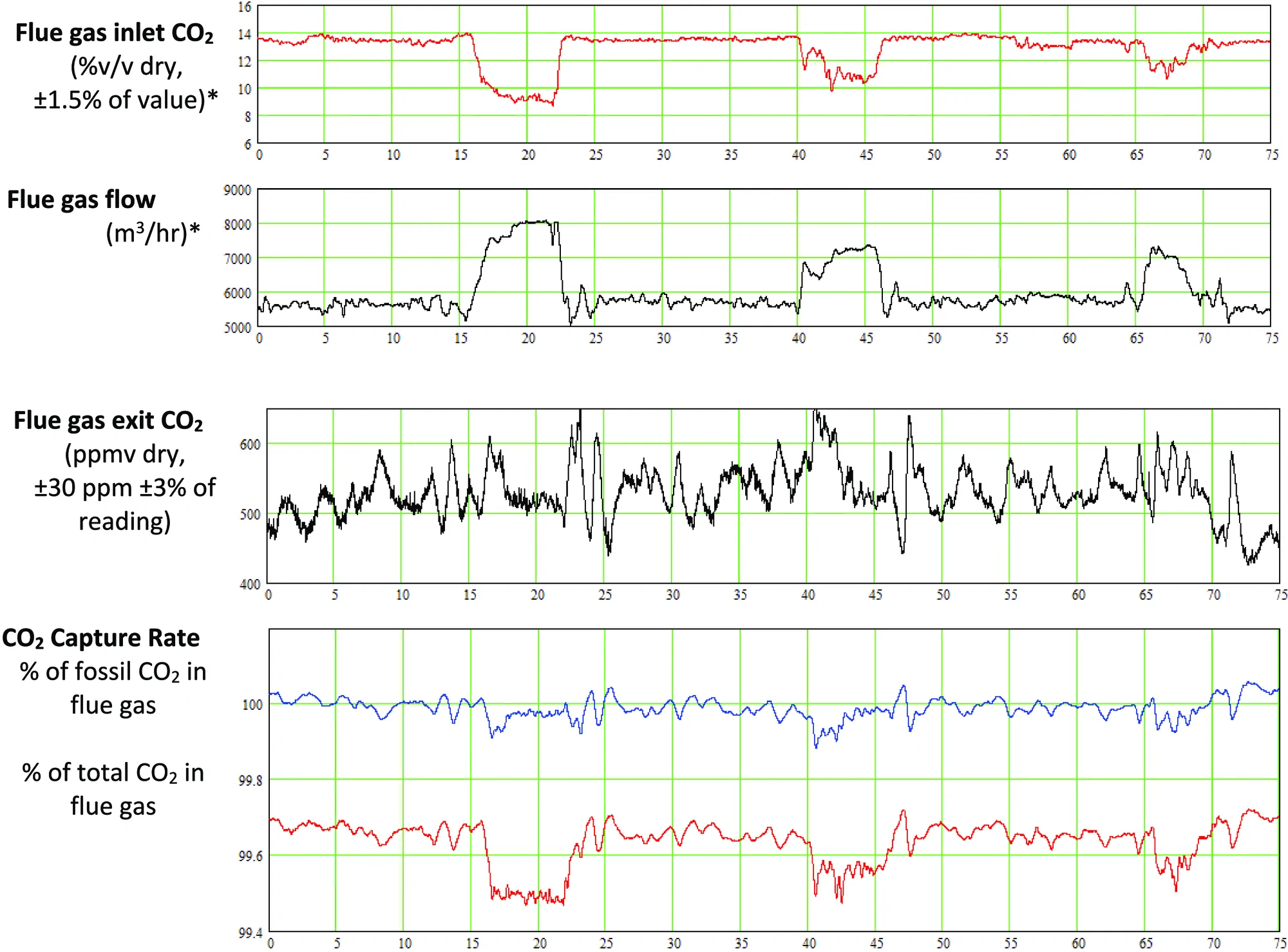
Absorber parameters over a 75 h period of active control.

As already discussed, in practical applications, a satisfactory absorber L/G ratio will have to be achieved by modulating the lean flow, with essentially uncontrolled incoming flue gas flow and CO_2_ concentration. As a result of the much greater average residence time in the absorber of solvent than that of flue gas, this may be more challenging than the flue gas flow control approach used in this work. Adequate absorber flow distribution, packing, and intercooling capacity for all gas/liquid flow combinations are also essential to enable satisfactory L/G matching.

Feedback control of the lean flow will be best achieved if there is a rapid indication of deviations from the required value. In this context, the observed time lag in these tests between changes in the total mass flow of CO_2_ to the absorber, due to changes in flue gas CO_2_ content and unintentional (in this case) variations in flue gas flow, was around 20 min, as shown in Figure 14. It is believed that this prolonged delay is due to the large amount of very lean solvent held up in the upper part of the absorber. For a time, this holdup ensures that near-equilibrium levels of exit CO_2_ are maintained, almost irrespective of the amount of CO_2_ rising from lower sections of the absorber.

**Figure 14:**
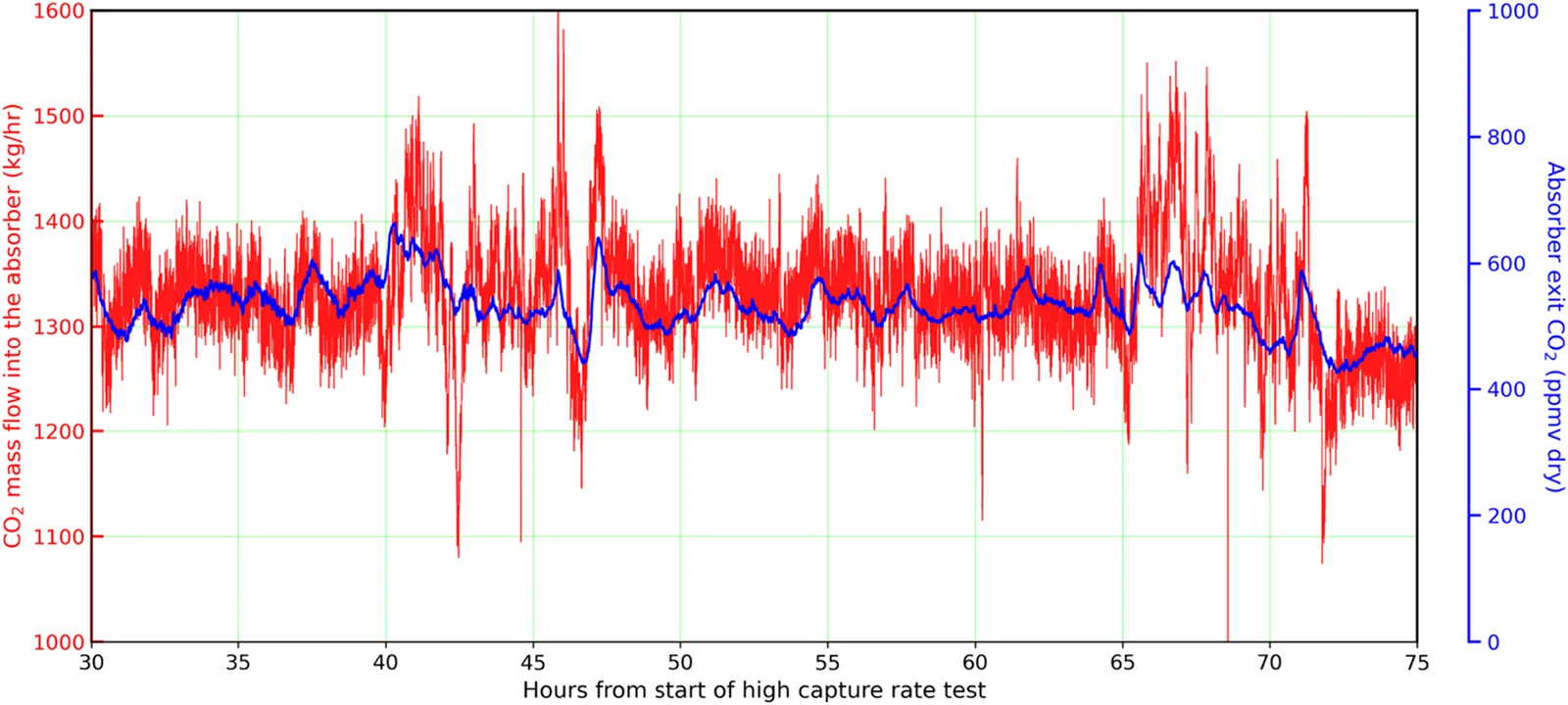
CO_2_ mass flow into the absorber (red, adjusted by controlling the flue gas flow in response to inlet CO_2_ concentration) and absorber exit CO_2_ (blue) 19.67 min later (best linear fit).

As shown in Figure 15, this delay in absorber CO_2_ exit concentration changes is actually slightly longer than the observed 18 min lag between changes in the mass flow rate of CO_2_ captured in the absorber, based on flue gas parameters, and the resulting changes in the CO_2_ flow leaving the desorber (with variation in the latter somewhat damped because of subsequent solvent mixing). This implies that changes in rich loading at the bottom of the absorber packing must be occurring with a time lag of under 18 min (and are also likely to be less damped than changes in the CO_2_ flow leaving the desorber). A real-time measurement of the rich loading leaving the desorber may therefore give a more rapid feedback signal for lean flow control than the absorber exit CO_2_ as well as being directly relevant to the achievable SRD.

**Figure 15:**
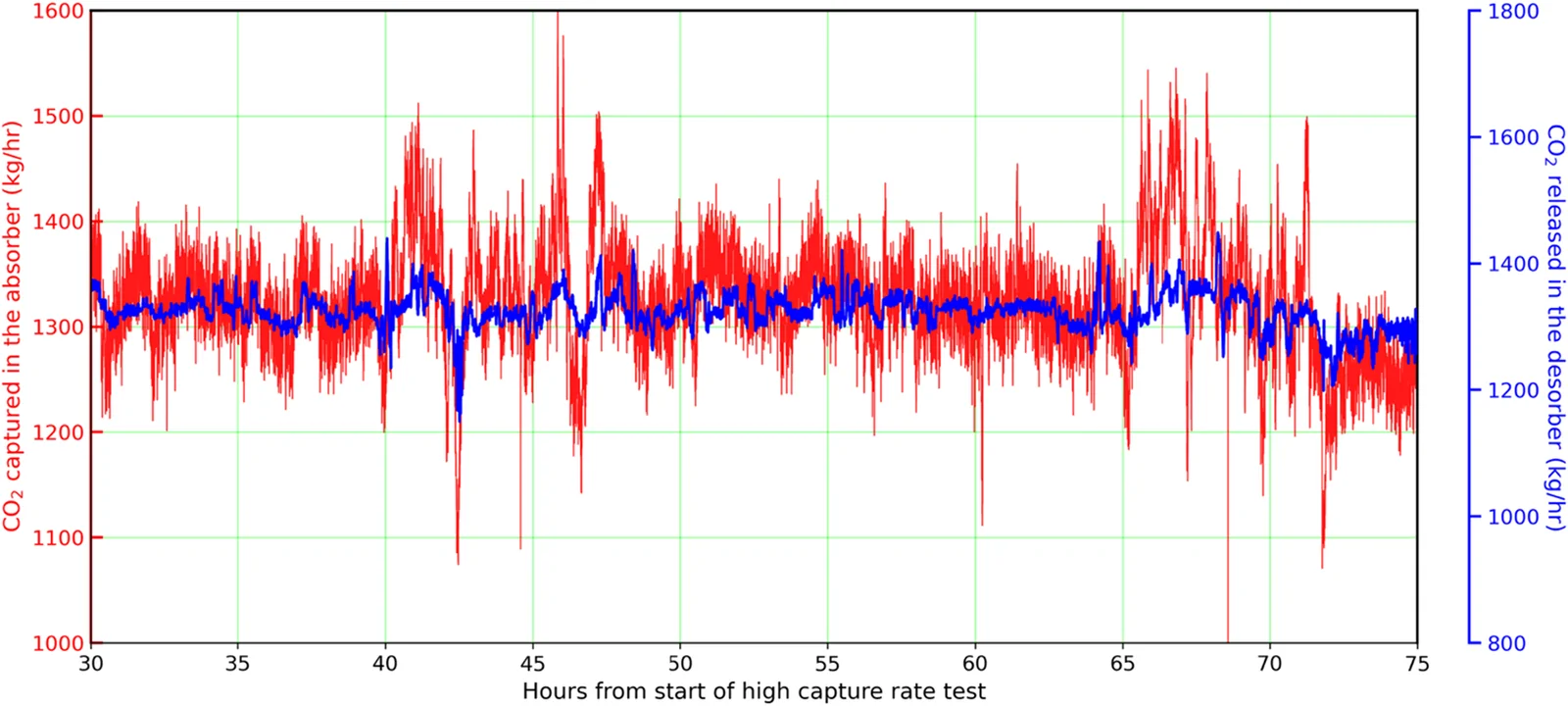
Calculated CO_2_ captured from flue gas (red,1289 kg/h average) and measured CO_2_ released in the desorber (blue, 1273 kg/h average) 18 min later (best linear fit).

For a possible way of achieving these rich loading measurements, Figure 16 shows time-series data collected during a short-term absorber test at the 1 tCO_2_/day EIC pilot facility at the University of Sheffield (using stored solvent^44^). Two rich loading measurements are shown: one based on density readings from a Coriolis flowmeter (Emerson Micro Motion F-series), downstream of the absorber sump, and another obtained from a trough collector probe located immediately below the absorber packing and feeding a density sensor (Wika Devil unit). Processing methods used to convert density measurements to loadings are described in Ibrahim et al.^49^ Also, by analogy with the proposed approach of calibrating the DBT target control value using the more representative, but slower responding, DCT value, it might be feasible to use the absorber exit CO_2_ value to continuously update the target rich density that is to be achieved.

**Figure 16:**
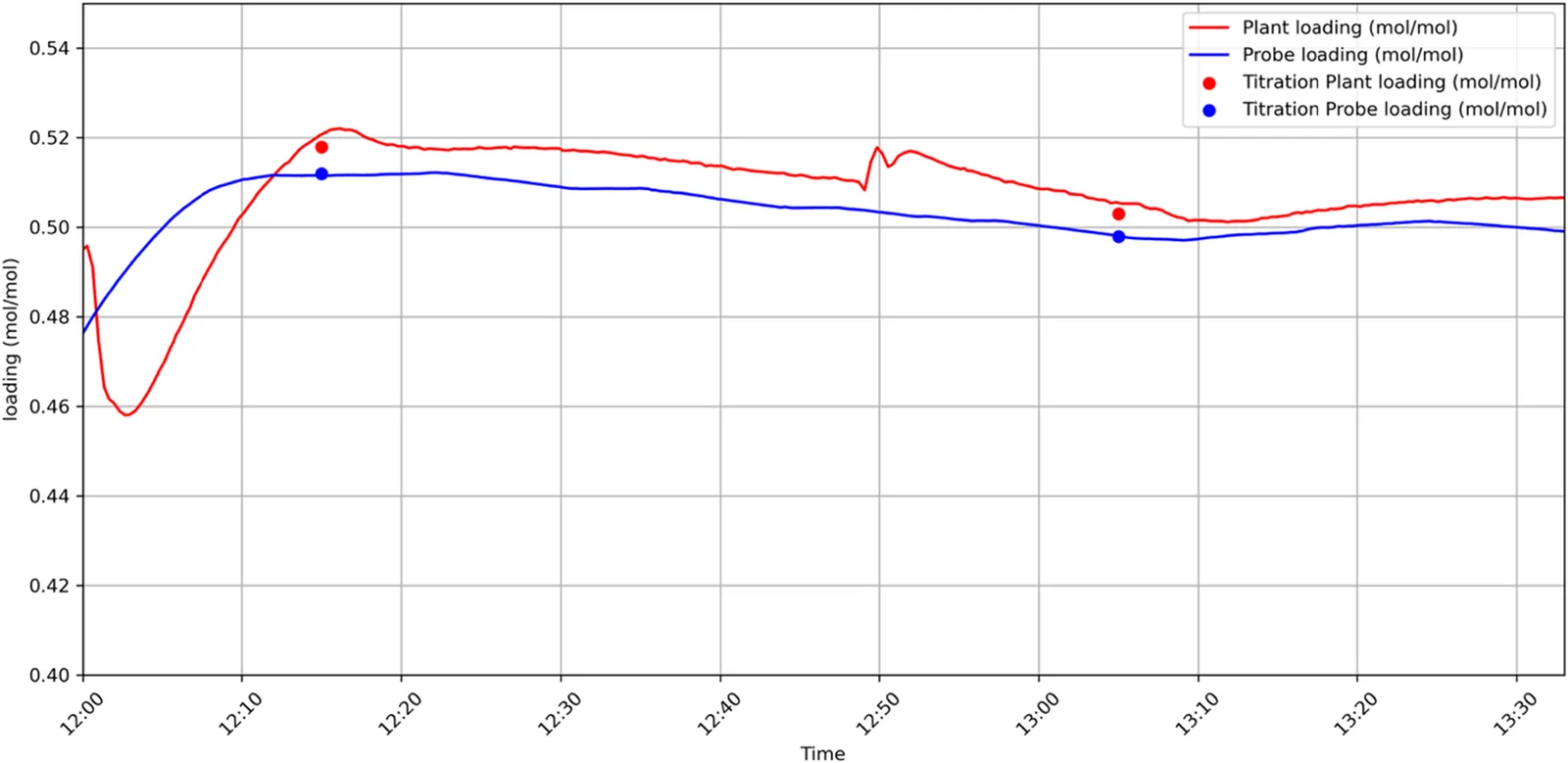
Example of absorber rich loading monitoring via density measurements.

In these EIC tests, the rich solvent loading values derived from the main plant Coriolis meter density readings exhibited a slower dynamic response and consistently higher values (confirmed by offline sample analysis). This is likely due to additional CO_2_ absorption occurring in the sump, tentatively attributed to gas bubble entrainment, although this behavior may be specific to the plant design. An additional excursion in the measured plant rich solvent loading values in Figure 16, which is not representative of absorber behavior, was caused by the addition of stored rich solvent to the absorber sump that had a different loading to the flow coming from the absorber packing.

These additional EIC pilot results suggest that data obtained using a rich solvent sampling probe situated immediately below the packing provide a faster and more representative measurement of real-time absorber performance than rich loading measurements taken downstream after the sump and any rich solvent storage. The measurements reported were, however, taken on a small pilot plant (absorber internal diameter 0.25 m) with a trough collector that spanned the full diameter. A probe system to collect a consistently representative rich solvent sample from a commercial-scale absorber would inherently be more complex. Consequently, there is merit in obtaining both a rich loading value using a point sample from an absorber packing exit probe and aggregated total flow rich loadings using measurements from a Coriolis meter handling the full rich flow downstream of the sump, as shown in Figure 16. The latter value can be used to cross-check against the former and represents the more relevant measurement for controlling the desorber performance. It may also be advantageous to assess the loading (using density measurement) for any intercooler streams or from other liquid collection systems between the absorber beds to provide advanced information on absorber operating point changes.

## 5 Conclusions

A novel manual feedback control method (with potential for automation) that allows amine post-combustion capture plants to operate at up to approximately 100% added CO_2_ capture has been developed from new process modeling insights. A version of this method has been tested in a pilot facility at a capture rate of 30 tCO_2_/day (10,000 tCO_2_/year). The tests were conducted using MEA, though the principles appear generally applicable to any ‘conventional’ amine capture system using a simple desorber. Although not conclusively shown in this paper, it also appears that the desorber likely needs to be fitted with a once-through thermosyphon reboiler, not a natural thermosyphon or similar reboiler linked to a large volume of retained lean solvent, to provide the fastest response to operator adjustments in reboiler steam input. This type of reboiler appears anyway to be favored for commercial PCC plants.

The additional instrumentation costs for implementing this method appear to be minimal. Desorber control is achieved using vapor phase temperature measurements below, above, and between the desorber packing beds to indicate when the desorber is at the inflection point, where the lowest possible lean loading is being achieved without excess water vapor in the product CO_2_. The accuracy of the temperature measurements is not at all critical because the temperature changes being observed at the center of the desorber packing as the desorber passes through the inflection point are very large. This also makes the approach insensitive to any ‘noise’ in the temperature data.

With the desorber operated to give essentially constant lean loading, the absorber control requirements then reduce to regulating the solvent flow to give the required exit CO_2_ level. This inherently requires sufficient precision and minimal noise in the exit gas CO_2_ analyzer reading. Supplementing this with a real-time indication of rich loading using solvent density measurements appears to be likely to further improve control. Utilizing a collection probe just below the packing provides the fastest possible response, in addition to measurements in the main rich transfer line. This setup helps to avoid inadvertently applying an incorrect L/G ratio in the absorber, an error that may not be immediately apparent from monitoring the exit CO_2_ concentration alone due to the slower and relatively small changes in exit CO_2_ with solvent flow at very high capture rates. This L/G imbalance may incur a lower rich loading and hence a higher regeneration energy penalty for little extra benefit in CO_2_ captured or, alternatively, an undesirably low capture rate.

Process modeling has been used to inform the formulation of the control method and to illustrate the ways in which it may work, but the control method is related primarily to the practical operation of amine capture plants rather than to their theoretical analysis. Experience on a large-scale pilot plant suggested that the control approach worked as expected, and details and ideas arising are therefore being shared to allow other operators to implement the method and determine whether similarly useful results can be obtained within the specific constraints of their own plant configuration.

The objective is to operate amine capture plants at around 100% capture indefinitely and, eventually, thus largely eliminate residual CO_2_ emissions from the CCS operation. A key requirement for this is the ability to sustain a high enough desorber pressure to deliver sufficiently low lean loadings at the desorber’s inflection point. Further long-term testing (of around 24 months) will be required to verify that thermal degradation at the associated reboiler temperatures (and with all other operating conditions representative of those that will be encountered in commercial practice) can be accommodated by suitable solvent management measures, but the control procedures described in this paper are a necessary prerequisite for these long-term tests.

## Supplementary Material



## Figures and Tables

**Figure 17:**
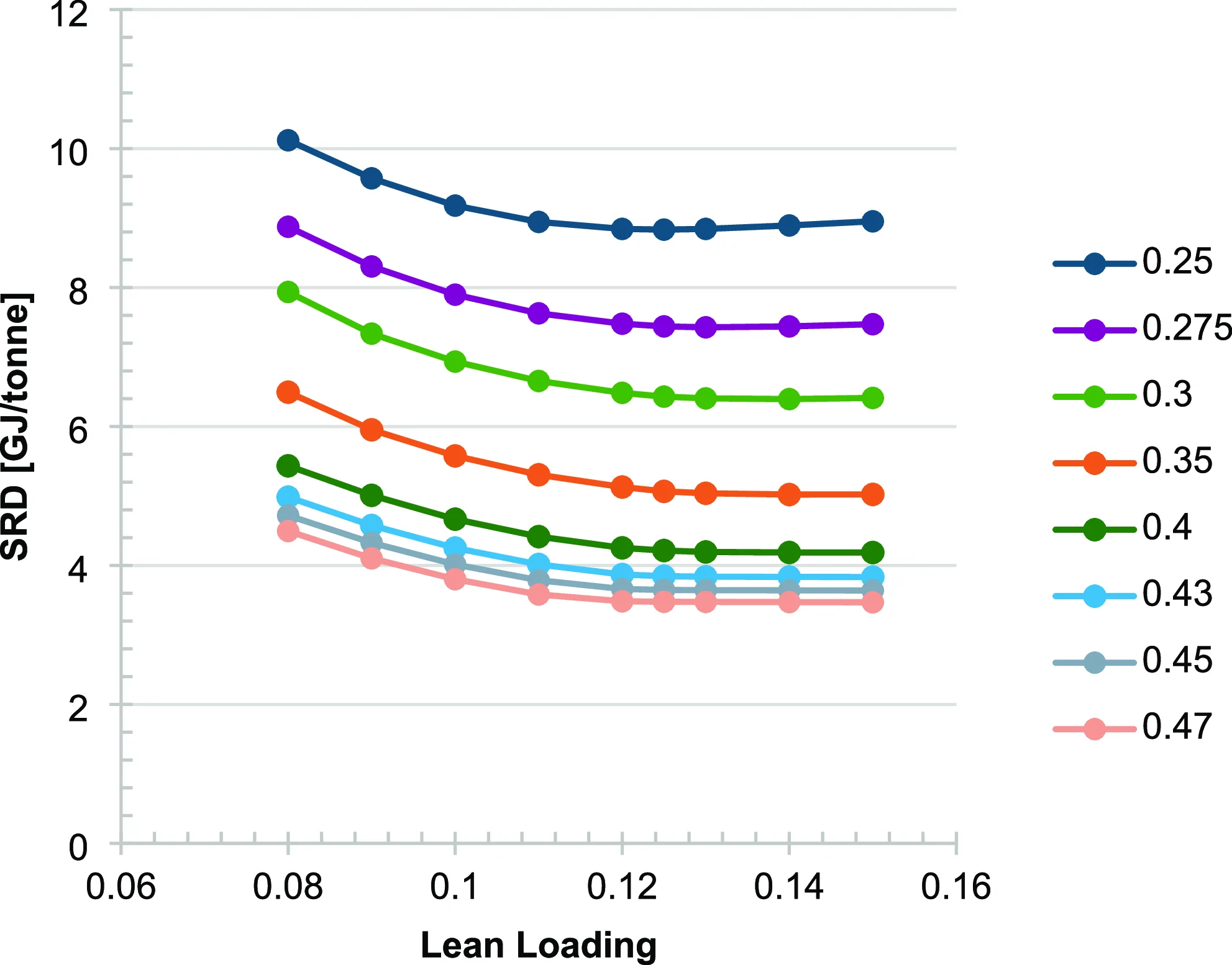
Desorber SRD for a range of constant rich loadings at an unloaded solvent concentration of 35 wt %.

**Figure 18:**
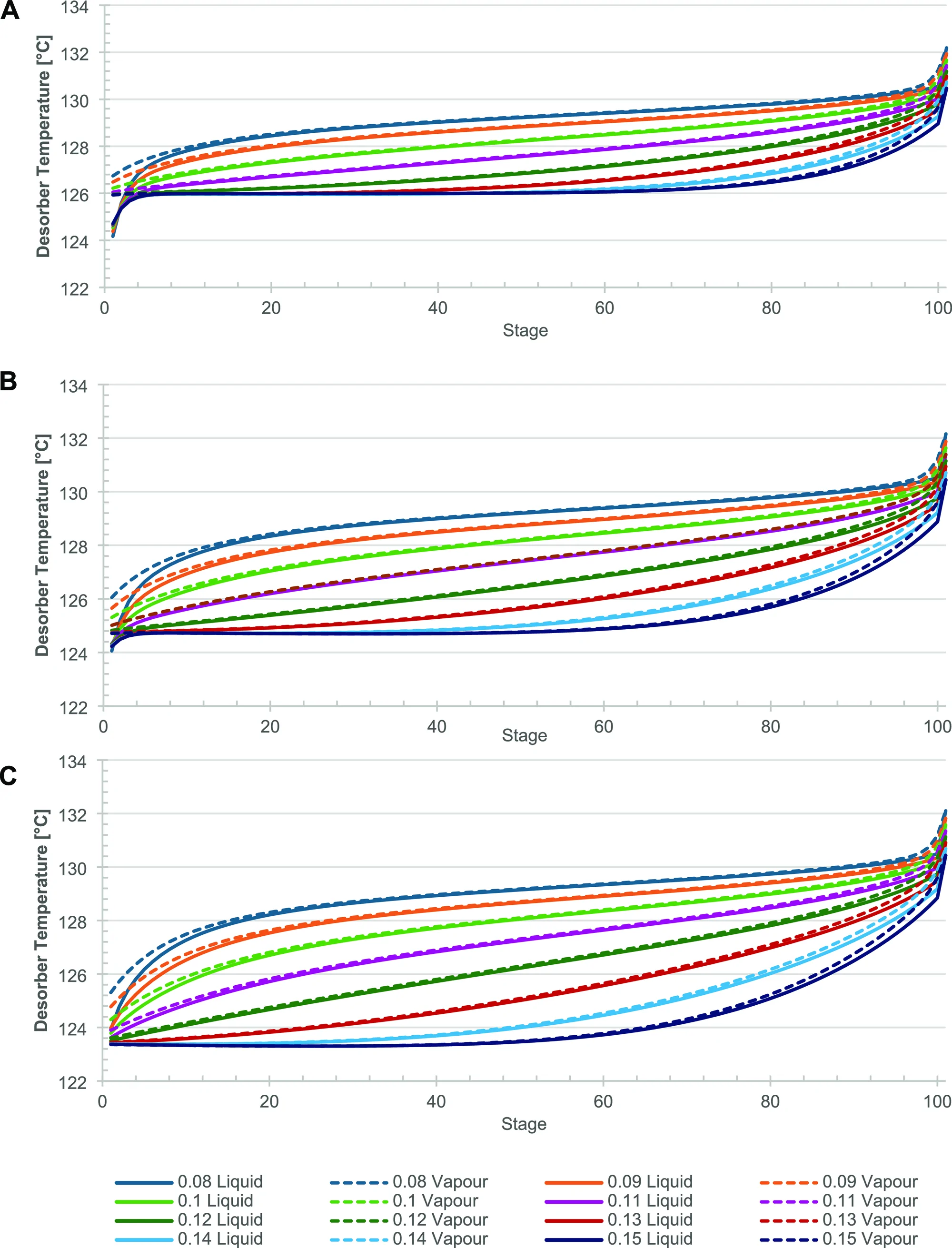
Desorber temperature profile of various lean loadings for a rich loading of 0.25 (A), 0.275 (B), and 0.3 (C) at 35 wt % MEA. The calculated vapor and liquid temperatures are shown with dotted and continuous lines, respectively.

**Figure 19:**
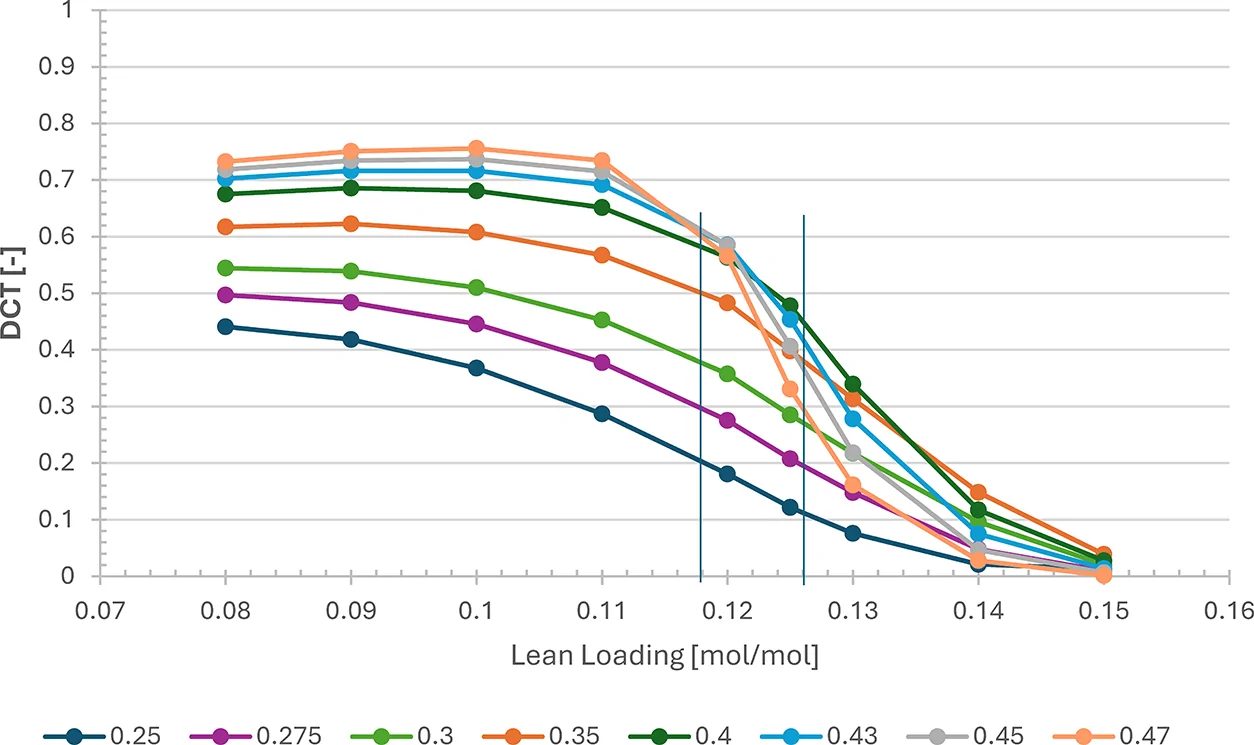
Dimensionless center temperature as a fraction of the desorber temperature rise for a range of lean loadings and rich loadings.

**Figure 20:**
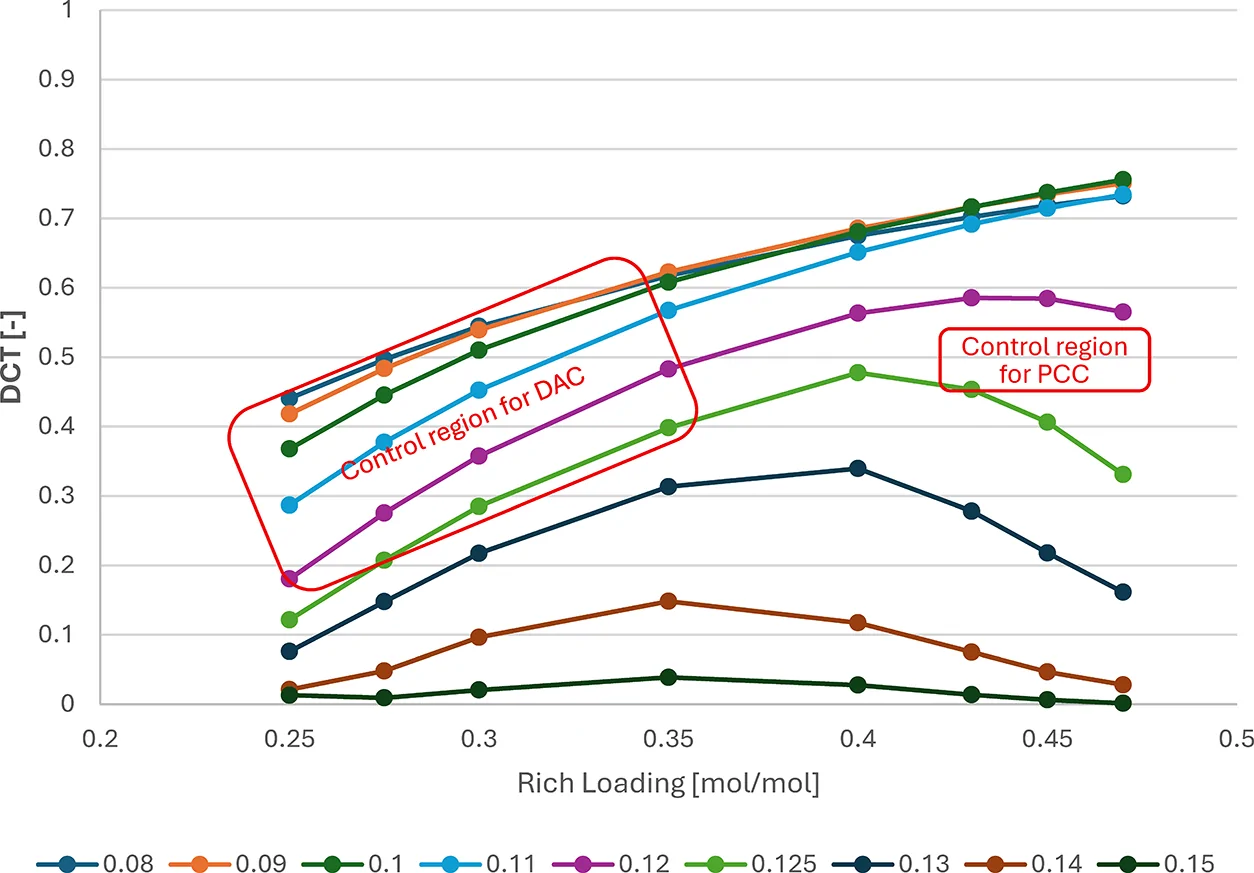
Dimensionless center temperature for different lean loadings as a function of rich loading—inflection point is between 0.12 and 0.125 lean loading.

## Appendix: A Preliminary Assessment of Using Desorber Temperature Profile Control at Low Rich Loadings

Rich loadings below 0.4 molCO_2_/molMEA are not expected to be viable in normal PCC operation because of the associated high SRD values. They may, however, be encountered in a PCC plant operating under start-up conditions, where minimum solvent flows to the packing are limited by flow distributor and packing wetting considerations and the flue gas flow and/or CO_2_ content may also be lower than in normal operation.

Low rich loadings will also arise with amine-based DAC units,^50^ with values of 0.3 molCO_2_/molMEA and lower, potentially limited by the low partial pressure of CO_2_ in the air. Because of the very high SRD penalty incurred if the solvent is regenerated at this low rich loading, as shown in Figure 17. It may therefore be preferable to subsequently use it in a flue gas absorber to raise the rich loading before regeneration,^5–52^ an arrangement termed CoDACCS.^53^ Even with CoDACCS installations, however, if there are extended periods when the flue gas source is not operating, there may be an economic case for operating the DAC plant in stand-alone mode, or a desire to maintain some CO_2_ flow in a transport and storage system.

Figure 17 shows desorber inflection point curves for 35% w/w MEA with rich loadings down to 0.25, with corresponding desorber temperature profile curves in Figure 18. Figure 19 shows the trends for dimensionless center temperature as a function of lean and rich loadings.

With lower rich loadings, the dimensionless center temperature value does not appear to plateau at lean loadings below the inflection point. As Figure 19 shows, if the rich loading is known, the dimensionless center point temperature could potentially be used to assess and control the delivered solvent lean loading at values down to around 0.08 molCO_2_/molMEA. Operation at these lower lean loadings is not expected to be attractive for flue gas capture, but it may be suitable for DAC, where it would significantly increase the driving force for mass transfer from the air. Furthermore, because the SRD is already quite high due to low rich loadings, the relative energy impact of operating at loadings below the inflection point is diminished.

Direct lean solvent loading measurements remain a viable alternative, but temperature measurements may be simpler to implement.

Figure 20 illustrates why a constant 0.5 dimensionless center temperature is a good approximation for PCC. It appears that DAC would require a target dimensionless center, or other packing location, temperature value based on both the achieved rich loading and the desired lean loading.

## Notes

Calculation methodology for Table 1 (PDF)

## Nomenclature

BECCS: biomass energy with carbon capture and storage
CCGT: combined cycle gas turbine
CCS: carbon capture and storage
CCSI: Carbon Capture Simulation Initiative
CDR: carbon dioxide removal (from the atmosphere)
CoDACCS: combined direct air capture and storage
DAC: direct air capture
DBT: desorber bottom temperature
DCT: dimensionless center temperature
Desorber: used synonymously for “stripper”
EA: Environment Agency https://www.gov.uk/government/organisations/environment-agency
EIC: Energy Innovation Center
e-NRTL: electrolyte non-random two-liquid (model)
GDCCUS: Guangdong CCUS (Center)
IEAGHG: IEA Greenhouse Gas Programme https://ieaghg.org/
IPCC: Intergovernmental Panel on Climate Change
L/G: liquid to gas (ratio)
MEA: monoethanolamine
NMPC: nonlinear model predictive control
NCCC: National Carbon Capture Center
PCC: post-combustion capture
ppmv: parts per million by volume (molar)
SRD: specific reboiler duty
TCM: Test Centre Mongstad
VLE: vapor liquid equilibrium
WECCS: waste to energy with carbon capture and storage
