# Supplementary material for: Principles and Practical Measurement and Control Strategies for Achieving Ultrahigh CO2 Capture Rates in Amine Post-Combustion Capture Plants
Source: Ind Eng Chem Res. 2026 Sep 23;65(39):21248–68. doi: 10.1021/acs.iecr.6c02191 (PMC13651545; doi:10.1021/acs.iecr.6c02191)
Supplement: Supplementary file 1 [file ie6c02191_si_001.pdf]

Supporting Information

Principles and practical measurement and control strategies for achieving ultra-high CO_2 capture rates in amine post-combustion capture plants

Lucas Joel[a], Aisha Ibrahim[a], Daniel Mullen[b], Abdulaziz Gheit[c], Muhammad Akram[c], Changyou Xia[d], Xi Liang[d], Qiang Jing[e], Weiming Zhao[e], Mathieu Lucquiaud[a] and *Jon Gibbins[a]

[a] School of Mechanical, Aerospace and Civil Engineering, The University of Sheffield, Sheffield, south Yorkshire, S3 7RD, United Kingdom

[b] SSE Thermal, One Forbury Place, 43 Forbury Road, Reading, RG1 3JH, United Kingdom

[c] Translational Energy Research Centre, The University of Sheffield, Sheffield, S9 1ZA, United Kingdom

[d] UK-China (Guangdong) CCUS Centre, 1 Tianfeng Road, Science City, Guangzhou 510520, China

[e] Shenzhen Shenshan Special Cooperation Zone China Resources Power Co., Ltd, Shenzhen 518066, China

*j.gibbins@sheffield.ac.uk

Methodology for calculations of 100% fossil CO_2 capture

1. **Coal**

Assuming fuel source is dry, ash-free, bituminous coal, the empirical formula is:

$$CH_{0.81}O_{0.08}S_{0.02}N_{0.01} \text{ [3]} \quad \text{[S1]}$$

Stoichiometric equation for combustion, assuming no CO_2 in the combustion air:

$$CH_{0.81}O_{0.08}S_{0.02}N_{0.01} + 1.165(O_2 + 3.76N_2) \rightarrow CO_2 + 0.405\ H_2O + 0.02\ SO_2 + 4.4583N_2 \quad \text{[S2]}$$

Excess Air: 15% [4]

Stoichiometric equation combustion of 1 mol of fuel with excess air, assuming presence of CO_2 in the inlet air:

$$CH_{0.81}O_{0.08}S_{0.02}N_{0.01} + (1.15\times1.165)\ (O_2 + 3.76N_2 + 0.002CO_2) \rightarrow 1.002719CO_2 + 0.405\ H_2O + 0.02\ SO_2 + 5.1196N_2 + 0.177375O_2 \quad \text{[S3]}$$

Assuming all SO_2 is removed in the stages before CCS, the flue gas concentration is calculated using the equation

$$\frac{\mathrm{nCO}_{2,f} + \mathrm{nCO}_{2,a}}{\mathrm{nCO}_2 + \mathrm{nN}_2 + \mathrm{nO}_2} = \frac{1 + 0.002719}{1.002719 + 5.116881 + 0.177375} = 1.59238 \times 10^{-1} = 159238\ ppmv \quad \text{[S4]}$$

Where $\mathrm{nCO}_{2,f}$ is the mols of fossil CO_2 (1), and $\mathrm{nCO}_{2,a}$ is the mols of atmospheric CO_2 (0.002719).

Capturing 100% of fossil-CO_2 leaves a residual amount, calculated as

$$\frac{\mathrm{nCO}_{2,a}}{\mathrm{nCO}_{2,a} + \mathrm{nN}_2 + \mathrm{nO}_2} = \frac{0.002719}{0.002719 + 5.116881 + 0.177375} = 5.13 \times 10^{-4} = 513\ ppmv \quad \text{[S5]}$$

The capture fraction is calculated using the equation

$$\frac{\mathrm{CO}_{2,in} - \mathrm{CO}_{2,out}}{\mathrm{CO}_{2,in}} = \frac{159238 - 513}{159238} = 99.68\% \quad \text{[S6]}$$

2. **BECCS**

Assuming fuel source has an empirical wood composition: $CH_{1.4}O_{0.6}$ [6]

Stoichiometric equation for combustion, assuming no CO_2 in the combustion air:

$$CH_{1.4}O_{0.6} + 1.05(O_2 + 3.76N_2) \rightarrow CO_2 + 0.7H_2O + 3.948N_2 \quad \text{[S7]}$$

Excess Air 30% [7]

Stoichiometric equation for combustion of 1 mol of fuel with excess air, assuming presence of CO_2 in the inlet air:

$$CH_{1.4}O_{0.6} + (1.05\times1.3)(O_2 + 3.76N_2 + 0.002CO_2) \rightarrow 1.00273CO_2 + 0.7H_2O + 5.1324N_2 + 0.315O_2 \quad \text{[S8]}$$

The flue gas concentration is calculated using the equation

$$\frac{nCO_{2,f} + nCO_{2,a}}{nCO_2 + nN_2 + nO_2} = \frac{1 + 0.00273}{1.00273 + 5.1324 + 0.315} = 1.55459 \times 10^{-1} = 155459\,ppmv \quad [S9]$$

Capturing 100% of fossil-CO_2 leaves a residual amount, calculated as

$$\frac{nCO_{2,a}}{nCO_{2,a} + nN_2 + nO_2} = \frac{0.00273}{0.00273 + 5.1324 + 0.315} = 5.01 \times 10^{-4} = 501\,ppmv \quad [S10]$$

The capture fraction is calculated using the equation

$$\frac{CO_{2,in} - CO_{2,out}}{CO_{2,in}} = \frac{155459 - 501}{155459} = 99.68\% \quad [S11]$$

3. WECCS

Assuming the fuel source has an empirical composition of: $C_{32}NH_{55}O_{16}$ [8]

The stoichiometric equation for combustion, assuming no CO_2 in the combustion air is:

$$C_{32}NH_{55}O_{16} + 37.75(O_2 + 3.76N_2) \rightarrow 32CO_2 + 27.5\,H_2O + 142.375\,N_2 \quad [S12]$$

Excess Air 190% [9]

Stoichiometric equation for combustion of 1 mol of fuel with excess air, assuming presence of CO_2 in the inlet air:

$$C_{32}NH_{55}O_{16} + (2.9\times37.75)(O_2 + 3.76N_2 + 0.002CO_2) \rightarrow 32.2188CO_2 + 27.5\,H_2O + 411.799\,N_2 + 71.725O_2 \quad [S13]$$

The flue gas concentration is calculated using the equation

$$\frac{nCO_{2,f} + nCO_{2,a}}{nCO_2 + nN_2 + nO_2} = \frac{32 + 0.2188}{32.2188 + 411.799 + 71.725} = 6.2471 \times 10^{-2} = 62471\,ppmv \quad [S14]$$

Capturing 100% of fossil-CO_2 leaves a residual amount, calculated as

$$\frac{nCO_{2,a}}{nCO_{2,a} + nN_2 + nO_2} = \frac{0.2188}{0.2188 + 411.799 + 71.725} = 4.52 \times 10^{-4} = 452\,ppmv \quad [S15]$$

The capture fraction is calculated using the equation

$$\frac{CO_{2,in} - CO_{2,out}}{CO_{2,in}} = \frac{62471 - 452}{62471} = 99.28\% \quad [S16]$$

4. CCGT

Assuming the fuel source is methane with an empirical composition of CH_4.

Also assuming that the flue gas CO_2 concentration including atmospheric excess dry air is 4.20 [5]. The excess air can be calculated by balancing the stoichiometric equation of combustion

The stoichiometric equation for combustion, assuming no CO_2 in the combustion air is:

$$CH_4 + (2\times X)(O_2 + 3.76N_2 + 0.002CO_2) \rightarrow (1+0.004\times X)CO_2 + 2\,H_2O + (7.52\times X)N_2 + (2\times X - 2)O_2 \quad [S17]$$

Where X is calculated to balance the equation such that

$$\frac{\mathrm{nCO}_{2,f} + \mathrm{nCO}_{2,a}}{\mathrm{nCO}_2 + \mathrm{nN}_2 + \mathrm{nO}_2} = 4.2 \times 10^{-2} \quad [S18]$$

This gives an excess air flow of 168.4%, balancing the stoichiometric equation to:

$$CH_4 + (2\times 2.684)(O_2 + 3.76N_2 + 0.002CO_2) \rightarrow 1.010736CO_2 + 2\,H_2O + 20.18368N_2 + 3.368O_2 \quad [S19]$$

The flue gas concentration is calculated using the equation

$$\frac{\mathrm{nCO}_{2,f} + \mathrm{nCO}_{2,a}}{\mathrm{nCO}_2 + \mathrm{nN}_2 + \mathrm{nO}_2} = \frac{1 + 0.010736}{1.010736 + 20.18368 + 3.368} = 4.2 \times 10^{-2} = 42000\,ppmv \quad [S20]$$

Capturing 100% of fossil-CO_2 leaves a residual amount, calculated as

$$\frac{\mathrm{nCO}_{2,a}}{\mathrm{nCO}_2 + \mathrm{nN}_2 + \mathrm{nO}_2} = \frac{0.010736}{1.010736 + 20.18368 + 3.368} = 4.66 \times 10^{-4} = 466\,ppmv \quad [S21]$$

The capture fraction is calculated using the equation

$$\frac{\mathrm{CO}_{2,in} - \mathrm{CO}_{2,out}}{\mathrm{CO}_{2,in}} = \frac{42000 - 466}{42000} = 99.89\% \quad [S22]$$

This information is available free of charge via the Internet at http://pubs.acs.org/
